# Discovery and preclinical characterization of the antagonist anti-PD-L1 monoclonal antibody LY3300054

**DOI:** 10.1186/s40425-018-0329-7

**Published:** 2018-04-30

**Authors:** Yiwen Li, Carmine Carpenito, George Wang, David Surguladze, Amelie Forest, Maria Malabunga, Mary Murphy, Yiwei Zhang, Andreas Sonyi, Darin Chin, Douglas Burtrum, Ivan Inigo, Anthony Pennello, Leyi Shen, Laurent Malherbe, Xinlei Chen, Gerald Hall, Jaafar N. Haidar, Dale L. Ludwig, Ruslan D. Novosiadly, Michael Kalos

**Affiliations:** 1 Lilly Research Laboratories, Department of Cancer Immunobiology, New York, NY USA; 2 Lilly Research Laboratories, Department of Preclinical Pharmacology, New York, NY USA; 3 Lilly Research Laboratories, Department of Biologics Technology, New York, NY USA; 4 Lilly Research Laboratories, Department of Non-Clinical Safety, Indianapolis, IN USA; 5 Lilly Research Laboratories, Department of Quantitative Biology, New York, NY USA; 6 Eli Lilly and Company, 450 East 29th Street, New York, NY 10016 USA; 7 Janssen Pharmaceutical Companies of Johnson and Johnson, Springhouse, PA USA

## Abstract

**Background:**

Modulation of the PD-1/PD-L1 axis through antagonist antibodies that block either receptor or ligand has been shown to reinvigorate the function of tumor-specific T cells and unleash potent anti-tumor immunity, leading to durable objective responses in a subset of patients across multiple tumor types.

**Results:**

Here we describe the discovery and preclinical characterization of LY3300054, a fully human IgG1λ monoclonal antibody that binds to human PD-L1 with high affinity and inhibits interactions of PD-L1 with its two cognate receptors PD-1 and CD80. The functional activity of LY3300054 on primary human T cells is evaluated using a series of in vitro T cell functional assays and in vivo models using human-immune reconstituted mice. LY3300054 is shown to induce primary T cell activation in vitro, increase T cell activation in combination with anti-CTLA4 antibody, and to potently enhance anti-tumor alloreactivity in several xenograft mouse tumor models with reconstituted human immune cells. High-content molecular analysis of tumor and peripheral tissues from animals treated with LY3300054 reveals distinct adaptive immune activation signatures, and also previously not described modulation of innate immune pathways.

**Conclusions:**

LY3300054 is currently being evaluated in phase I clinical trials for oncology indications.

**Electronic supplementary material:**

The online version of this article (10.1186/s40425-018-0329-7) contains supplementary material, which is available to authorized users.

## Background

T cell activation occurs when T-cells receive two positive signals from antigen-presenting cells (APC): an antigen-specific signal presented in the context of major histocompatibility complex (MHC) which engages the T-cell receptor (TCR), and a co-stimulatory signal from B7–1/B7–2 (CD80/CD86) to the CD28 receptor on T-cells [1]. Initial T cell activation is followed by the surface expression of a set of co-activating receptors such as CD137, OX40, GITR, and CD27 which enhance T-cell function, and a set of T-cell inhibitory receptors which initiate inhibitory pathways that function to prevent uncontrolled T-cell proliferation and function, and ultimately restore T-cell functional homeostasis [2]. The prototypic T-cell inhibitory (i.e. “checkpoint”) receptors are CTLA-4 (CD152) and PD-1 (CD279), and the regulatory approval of agents that target CTLA-4 (ipilimumab, Yervoy™), and PD-1 (nivolumab (Opdtivo™), pembrolizumab (Keytruda™), has been key to bringing forth the modern era of immunotherapy.

Two ligands have been described for PD-1: PD-L1 ((B7-H1, CD274), and PD-L2 (B7DC, CD273). While baseline expression of PD-L2 is relatively limited to subsets of dendritic cells, macrophages, B cells, mast cells and Th2 cells and tumor cells [3], expression of PD-L1 is substantially broader with expression by APC, myeloid cells, subsets of activated T cells, endothelium, as well as a broad range of tumors (reviewed in [4–6]). While one physiological role of PD-L1 is believed to involve the suppression of T-cell activation to minimize damage to normal tissues by activated T cells [7, 8], more recent evidence suggests that PD-L1 might also play important roles to modulate innate immunity by sensing hypoxic [9] and metabolic [10] stress. PD-L1 also binds to a second receptor B7–1 (CD80), which is the inhibitory ligand for CTLA-4 and is expressed on dendritic cells, macrophages, activated T and B cells and some non-hematopoietic cells (liver stromal cells and keratinocytes) [6], raising the to-date untested possibility that the PD-L1 ligand may play a role to modulate both the PD-1 and CTLA-4 T cell inhibitory pathways.

The PD-L1/PD-1 axis is often subjugated by tumors to evade anti-tumor immune response; indeed, PD-L1 expression in tumor tissues has been an important predictive biomarker of response for PD-1 pathway inhibitors across multiple cancers and molecules in clinical development. PD-L1 is genetically dysregulated in a variety of tumor types, and increased expression of PD-L1 by tumors correlates with a poor prognosis in patients with lung, ovarian, renal and other solid tumors [11–13]. PD-L1 expression can also be up-regulated in the tumor microenvironment as a result of immune activation and production of pro-inflammatory cytokines such as interferon-gamma (IFNγ), contributing to the establishment of an “adapted” T-cell immunosuppressive milieu [14].

The clinical validation of targeting the PD-1/PD-L1 axis demonstrated by inhibition of the PD-1 receptor, has also led to the clinical development and regulatory approval of multiple molecules that block the PD-L1. To-date this list of approved PD-L1-targeting agents includes atezolizumab (Tecentriq™), avelumab (Bavencio™), and durvalumab (Imfinzi™) across multiple tumor types and lines of therapy (reviewed in [15]). Approved antibodies that target the PD1/PD-L1 axis include both effector competent and Fc effector-ablated molecules, without a to-date clear picture about how this variable might impact activity in the clinical setting.

Despite the to-date approval of a number of medicines that target the PD-1/PD-L1 axis, considerable efforts to develop additional agents that target this pathway are ongoing both clinically and pre-clinically, with multiple agents currently at various stages of development; these efforts reflect the broad recognition and acceptance that targeting the PD-1/PD-L1 axis is likely to be a foundational component for future immunotherapy-based strategies to treat cancer.

Analysis of the PD-1/PD-L1 co-complex structure has revealed a protein-protein interface that is largely devoid of deep pockets [16], an interface typically amenable to targeting by antibodies. High resolution crystallographic data sets have demonstrated that the epitopes of the PD-1 blocking antibodies nivolumab and pembrolizumab structurally cluster on the flat PD-L1-binding surface of PD-1 and overlap with the binding site for PD-L1 [17–19]. While no structural data is publically available for the crystal complexes of atezolizumab, durvalumab, or avelumab with PD-L1, the epitope of avelumab and the anti-PD-L1 antibody BMS-93559 have been also shown to structurally overlap the binding site for PD-1 on PD-L1 [19, 20].

To-date preclinical development of agents that target the PD-1/PD-L1 axis has been based on the use of in vitro human assay systems which have revealed the potential for blockade of PD-1 and PD-L1 to increase T-cell activation and function, and in vivo preclinical experiments with surrogate, murine-specific antagonist antibodies and syngeneic immune competent mouse models. These models have demonstrated that blocking the murine PD-1/PD-L1 axis can result in immune-mediated anti-tumor activity and in a number of cases cooperativity and/or synergy with other T cell modulating agents [21–23]. However, these studies have largely failed to explore the functional and mechanistic properties of the clinical agents on human immune cells, including understanding how blockade of the PD-1/PD-L1 axis might impact immune functions beyond T cells. More recently, an anti-PD-1 antibody (REGN2810) has been characterized in a mouse model with human PD-1 knock-in, and shown to enhance anti-tumor murine T cell immunity in that setting [24].

Here we describe the discovery, characterization, and preclinical development of LY3300054, an antagonist anti-human PD-L1 antibody isolated from a human ScFv phage display library. LY3300054 is a fully human IgG-1 antibody engineered with ablated Fc-mediated immune effector functions. LY3300054 potently blocks interaction of PD-L1 with its cognate receptors PD-1 and CD80, and cross-reacts with cynomolgous PD-L1. Sequence alignment and mutagenesis demonstrates that residue N63 on PD-L1, which is part of the PD-L1/PD-1 interphase, is a key residue for the target and species specificity of LY3300054. LY3300054 is capable of modulating T cell effector functions in a variety of in vitro immune cell functional assays, and to enhance T cell functional activation and T cell-mediated anti-tumor activity in three different mouse xenograft tumor models with reconstituted human immune cell compartments. High-content molecular analysis of tumor and peripheral tissues collected from these animals reveals a broad spectrum of immune-related intra- and extra-tumoral effects for LY3300054, including previously not described effects on innate immune pathways. LY3300054 is currently under clinical evaluation in monotherapy and combination with other therapeutic modalities in multiple tumor types (NCT02791334; NCT03099109; NCT02791334; NCT02791334).

## Methods

### Phage screening

A human scFv phage display library (AbCheck, Czech Republic) was used to identify phage antibodies that bound recombinant human PD-L1-Fc protein. Phage that bound to human Fc, CD80 and CD86 were depleted from the libraries by pre-incubation steps throughout the panning process. In some cases, libraries were heated to 65 °C for 15 min prior to the panning step to select for heat-stable scFv. Enrichment of PD-L1 specific scFv was tested with bacterial extracts containing soluble scFv in ELISA. Panned phages were screened for the presence of scFv that blocked the interaction of PD-L1 with both PD-1 and CD80. Clone ABC110 (LY3300054) was selected from a large number of functional hits based on binding, blocking, and in-vitro functional properties, and its DNA sequence was cloned into a human expression vector with an IgG1 effector-null backbone (IgG1-EN), containing the following residue changes; L234A, L235E, G237A, A330S, and P331S (11520463), and CHO cells that stably expressed LY3300054 were established. LY3300054 IgG was purified from the culture supernatant by protein A affinity chromatography (Poros A, Applied Biosystems, Foster City, CA). By flow cytometry LY3300054 was shown to specifically bind to the surface of the PD-L1-positive (H292, HCC827) but not the PD-L1–negative A204 cell lines (Additional file 1: Figure S1).

### Protein expression and purification

The extracellular domain (ECD) of human PD-L1 was cloned into an Fc (human IgG1) construct (GS vector) that contained a Factor Xa cleavage site at the N-terminus of the hinge region. Human PD-L1-Fc was expressed in human 293-Freestyle cells (Invitrogen Corp., Carlsbad, CA) that were cultivated and transfected according to manufacturer’s specifications. Human PD-L1-Fc was purified via standard ProA affinity columns; human PD-L1 monomer was cleaved from the purified Fc construct with Factor Xa enzyme. Cleaved Fc and undigested PD-L1-Fc were purified out of the sample via standard ProA affinity column. Purified proteins were buffer exchanged into PBS, quantified and evaluated by SDS-PAGE and analytical SEC analysis to confirm structural integrity. Canine PD-L1-Fc and its mutants were expressed transiently in Expi293F cells following transfection using ExpiFetamine 293. The canine PD-L1-Fc and its mutants in addition to the cynomolgus, murine and rat PD-L1-Fc were generated in a manner similar to that of the human PD-L1-Fc.

### ELISA binding assays

#### Binding to recombinant PD-L1

Ninety-six-well plate (Immulon 2HB) was coated with 100 ng of human PD-L1-Fc, murine PD-L1-Fc, or cynomolgus PD-L1-Fc (R&D Systems, Minneapolis, MN) overnight at 4 °C. Wells were blocked for 2 h with blocking buffer (PBS containing 5% nonfat dry milk) and then washed three times with PBS containing 0.1% Tween-20. 100 μl of serially diluted anti-PD-L1 antibody or control IgG was then added and incubated at room temperature for 2 h. After washing, the plate was incubated with goat anti-human IgG F(ab’)2-HRP conjugate (Jackson ImmunoResearch, West Grove, PA) at room temperature for 1 h. The plates were washed and then incubated with 3, 3′, 5, 5′-tetramethylbenzidine. The absorbance at 450 nm was read on a microplate reader. The half maximal effective concentration (EC50) was calculated using GraphPad prism software.

#### Binding to canine PD-L1 variants

Ninety-six well Immulon 4HBX ELISA plate was coated overnight with 50 ng each of the wild type and mutant canine PD-L1-ECD-Fc in 100 μl of PBS, pH 7.2 with mild agitation at 4 °C. After blocking and wash, a five-fold dilution series (0.0017–133 nM) of LY3300054 was added in duplicate and incubated with mild agitation for 1 h at room temperature. The wells were washed and a 1:10,000 dilution of HRP-conjugated goat anti-Fab antibody (Jackson ImmunoResearch) was added and incubated at room temperature following standard protocol. TMB peroxidase chromogenic substrate and stop solution were used according to manufacturer’s instruction for visualization and detection of signals. Absorbance readings were plotted in GraphPad Prism software. EC_50_ values were calculated by nonlinear regression curve fit analysis of the software’s One Site-Specific Binding function.

### ELISA blocking assays on PD-L1 interaction with PD-1 or CD80

Serially diluted LY3300054 or control IgG were mixed with the equal volume of a fixed concentration of biotinylated PD-L1-Fc (100 ng/mL for PD-1 blocking and 500 ng/mL for CD80 blocking), and then incubated at room temperature for 1 h. 100 μl of the mixture was transferred to 96-well plates pre-coated with human PD-1-Fc or with human CD80-Fc at 100 ng/well (R&D Systems) and then incubated at room temperature for an additional 1 h. After washing, Streptavidin-HRP conjugate was added, and absorbance at 450 nm was read. IC50 represents the antibody concentration required for 50% inhibition of PD-L1 binding to PD-1 or to CD80.

### SPR binding to recombinant human, murine or cynomolgus PD-L1

Surface plasmon resonance (SPR) (Biacore T200, GE Healthcare) was used to determine the binding kinetics of LY3300054 to human, cynomolgus, murine and rat PD-L1-Fc at 37 °C. Approximately 40 response units (RU) of LY3300054 were immobilized onto a CM5 chip using the standard amine coupling procedure. HBS-EP buffer (10 mM HEPES, 150 mM NaCl, 3 mM EDTA, and 0.005% surfactant p20) was utilized as a running buffer during binding kinetic measurements. The PD-L1-Fc gradients were comprised of seven 3× dilutions. Starting concentrations were 9 nM for the human and cynomolgus PD-L1-Fc gradients and were 90 nM for the mouse and rat PD-L1-Fc. PD-L1-Fc proteins were injected for 180 s (contact time) over the immobilized LY3300054 at a flow rate of 30 μl/min. The dissociation times for those measurements were 1500 s for the four top concentrations of the gradient and 240 s for the rest of the gradient. After dissociation, regeneration of the LY3300054 surface was achieved with a single 18 s injection of 0.75 M NaCl/25 mM NaOH at 30 μl/min followed by a 30 s wash with HBS-EP to stabilize the surface. Biacore T200 Evaluation Software (version 1.0) was used to analyze the results from the kinetic experiments. After double referencing to remove artifacts from nonspecific binding, simultaneous global fitting of the data for each concentration gradient to a 1:1 L model was performed to determine the association rate (*k*_on_), dissociation rate (*k*_off_), and dissociation constant (*K*D = *k*off/*k*on). At least four different concentration gradients were used to compute the kinetic parameters and their corresponding sample standard deviation.

### In vitro functional assays

#### PD-1 reporter assay

PD-L1^+^ aAPC/CHO-K1 (Promega) or PD-L1^−^ aAPC/CHO-K1 (Promega part# CS187110) human T-activator cells were plated in a 96-well white opaque tissue culture plate at 40,000 cells per well in 100 μl of medium (10% FBS F-12, 0.2 mg/ml Hygromycin-B and 0.2 mg/ml G418) and incubated overnight at 37 °C at 5% CO_2._ Medium was removed from the assay plate the following day and serially diluted test and control antibodies were added at 40 μl per well in the assay buffer. GloResponse NFAT-luc2/PD1 Jurkat cells (Promega) were re-suspended in assay buffer at a concentration of 1.25 × 10^6^ /ml and added to the plate at 40 μl per well. After 6 h of co-culture, assay plates were removed from the incubator and equilibrated at room temperature for 5 min. Bio-Glo™ Reagent (Promega) was prepared according to manufacturer’s instructions and added to each well at 80 μl per well. Plates were then incubated for 5 min at room temperature. Luminescence was measured in a plate reader and data was analyzed using GraphPad Prism software [25].

#### Mixed leukocyte reaction (MLR)

CD14^+^ monocytes were isolated from frozen human peripheral blood mononuclear cells (PBMC) obtained from a healthy donor (AllCells, Alameda, CA) with Human Monocyte Isolation Kit II (Miltenyi, Auburn, CA). Immature dendritic cells (DCs) were generated by culturing these monocytes in complete RPMI-1640 medium containing 10% FBS in the presence of 1000 IU/ml hGM-CSF and 500 IU/ml hIL-4 for 4 days. CD4^+^ T cells were purified from fresh human PBMC of a different healthy donor (AllCells) using Human CD4^+^ T Cell Isolation Kit (Miltenyi). The two types of cells were then mixed in 96-well V-bottom plates with 5 × 10^4^ CD4^+^ T cells and 5 × 10^3^ immature DC in 100 μl of complete AIM-V medium per well. 100 μl of 2× serially diluted LY3300054 or human IgG1 was added into a well of the plates. LY3300054 was also tested in combination with anti-CTLA4 antibody (Ipilimumab) at equimolar concentrations ranging from 0.003 to 67 nM. After incubation for 72 h at 37 °C at 5% CO2, supernatants and cell pellets were harvested and subjected to immunoassay (human IFN-γ ELISA (R&D Systems) or 41-plex Milliplex MAP Human Cytokine/Chemokine Immunoassay Panel (Millipore, Burlington, MA) (analytes are listed in Additional file 2) and a custom-made Quantigene Plex gene expression analysis (see below). MLR studies of LY3300054 were repeated with at least four different CD4 T cell donors.

#### Antigen recall assay

Frozen PBMCs were thawed, cultured in 10% FBS RPMI overnight at 37 °C at 5% CO_2_, and seeded in a 96-well flat bottom tissue culture plate at 1 × 10^5^ cells per well in 100 μl of 10% FBS/RPMI-1640. Antibodies were prepared at 4× concentrations and added to the cells at 50 μl per well. After 1-h incubation, Tetanus Toxoid (50uL; 0.8μg/ml) (TT; #191A LIST Biological Laboratories Inc.) was added to wells with LY3300054 or medium control. After 5 days in culture, supernatant was collected and an IFNγ ELISA (R&D Systems SIF50) was performed according to manufacturer’s instructions.

### Effector function assays

#### Antigen-dependent cell-mediated cytotoxicity (ADCC) assay

The ability of LY3300054 to mediate ADCC was tested in a Jurkat-FcγRIIIa reporter gene assay using a PD-L1^+^ HEL cell line (ATCC TIB-180) as previously described [26]. Anti-CD20 antibody rituximab (wild type IgG1) was tested as a positive control in the same assay against the CD20-positive WIL2-S cell line. Briefly, 1 × 10^4^ target cells at 50 μl and serially diluted antibodies at 4× concentrations at 25 μl were added per well. Jurkat-FcγRIIIa (V158) cells were added as effector cells at the effector/target ratio of 15:1 at 25ul/well, and followed by 6 h incubation in a humidified 37 °C incubator. Plates were removed and equilibrated to room temperature for 5 min. Luciferase reagent was added at 100 μl/well and luminescence was detected.

#### Complement dependent cytotoxicity (CDC) assay

LY3300054 was tested using the PD-L1^+^ HEL cells as targets. Rituximab was used as a positive control against WIL2-S cell line in the same experiment. Target cells were treated with 1:3 titrations of the various antibodies and incubated for 30 min at 37 °C. Human complement was added into the assay plates and incubated for 1 h at 37 °C. Alamar Blue reagent was then added to the wells and incubated for an additional 24 h at 37 °C before fluorescence was determined, as an indication of cell viability.

### PBMC cytokine release assay

Fresh unstimulated human PBMC isolated from six healthy donors were incubated with plate bound LY3300054 antibody or control antibodies for 24 h, pre-coated over a broad titration range from 0.003 to 100 μg/ml. Anti-CD3 antibody OKT3 (eBioscience, San Diego, CA) was used as a positive control. Using a commercially available multiplex assay based on the Luminex platform (Luminex Corporation, Austin, TX), 21 cytokines including Fractalkine, GM-CSF, IFNγ, IL-1β, IL-2, IL-4, IL-5, IL-6, IL-7, IL-8, IL-10, IL-12 (p70), IL-13, IL-17A, IL-21, IL-23, ITAC, MIP-1α, MIP-1β, MIP-3α, and TNF-α were measured in cell culture supernatants [27].

### PD-L1 and HLA class I staining of human tumor lines

NCI-H292, HCC827, OV79, and A204 (ATCC) tumor cells were cultured for approximatelly36 hours prior to non-enzymatic harvest. NCI-H292, HCC827, and A204 cells were stained for PD-L1 using FITC-conjugated anti-human PD-L1 commercial antibody (clone MIH1, BD Biosciences), Alexa Fluor® 488-conjugated LY3300054, or appropriate isotype controls. NCI-H292, HCC827, and OV79 cells were stained separately for HLA Class I expression using an APC-conjugated antibody (clone W6/32, RnDSystems, Minneapolis, MN) Samples were collected on a 5-laser Fortessa X-20 cytometer (BD Biosciences) and analyzed with FlowJo V10 software (TreeStar).

### In vivo models

All animal studies were approved by the Institutional Animal Care and Use Committee and performed in accordance with current regulations and standards of the United States Department of Agriculture and the National Institute of Health. All experiments with adoptively transferred human PBMC or expanded human T cells utilized NOD.*Cg-Prkdc*^*scid*^*Il2rg*^*tm1Wjl*^/SzJ (NSG) animals (6–7 weeks of age, female, from Jackson Laboratories, Bar Harbor, MN), and were maintained in a 12 h light/dark cycle facility under pathogen-free conditions in microisolator cages with standard laboratory chow and water ad libitum. Cord blood-derived CD34^+^ hematopoietic stem cell (HSC) engrafted mice used for the OV79 model utilized NOD.Cg-*Prkdc*^*scid*^ *Il2rg*^*tm1Sug*^/JicTac animals (NOG, 15–17 weeks of age, female) and were obtained from Taconic BioSciences (Rensselaer, NY). Fetal liver derived CD34^+^ HSC transplanted mice used for the HCC827 model in NSG background (15–17 weeks of age, female) were obtained from Jackson Laboratories. Animal well-being and behavior, including grooming and ambulation were monitored at least twice per week. Body weight and tumor volumes were measured twice a week starting 1–2 weeks post implantation. Tumor volumes were calculated according to formula (vol = π/6 * l * w^2^) and plotted as geometric means ± standard error of the mean (SEM). Statistical analysis of tumor volume data was performed by two-way ANOVA on repeated measurements.

#### Co-implantation of human NCI-H292 tumor cells and human PBMC (Winn model)

Freshly isolated human PBMCs were combined with freshly cultured NCI-H292 tumor cells (ATCC, Manassas, VA) at a 1:4 E:T ratio and co-implanted subcutaneously into the flanks of female NSG mice (groups of 8 mice per treatment arm). One day later, weekly intraperitoneal (IP) treatments of either human IgG1 or LY3300054 at 10 mg/kg began and continued for a total of four doses. Tumor growth was monitored by caliper measurements.

#### Established HCC827 xenograft tumor model with infused human T cells

Mice were implanted subcutaneously into the flanks of female NSG mice with 10 × 10^6^ freshly cultured HCC827 tumor cells (ATCC). When tumors reached volumes of ~ 300 mm^3^ (~ 4–5 weeks), 2.5 × 10^6^ expanded human T cells were administered intravenously (IV) and mice were treated with weekly IP injections of human IgG1 or LY3300054 at 10 mg/kg for a total of four doses.

Established xenograft tumor models in CD34^+^ hHSC-engrafted mice: Cord blood derived CD34^+^ hHSC transplanted NSG mice were implanted subcutaneously with serially passaged HCC827 tumor fragments (4–5 mm in diameter) at 15–17 weeks of age. When the tumors reached volumes of approximately 200 mm^3^ (~ 30 days), weekly IP treatments of human IgG1 or LY3300054 at 10 mg/kg began for a total of three doses. Fetal liver-derived CD34^+^ hHSC transplanted NOG mice were implanted subcutaneously with serially-passaged OV79.FFluc2A–gfp tumor fragments (4–5 mm in diameter) at 15–17 weeks of age. OV79.FFLuc-2A-gfp tumor cells are an ovarian carcinoma line transduced with lentivirus encoding firefly luciferase and green fluorescent protein from a bicistronic transcript [28] and will be hereafter be referred to as OV79. When tumor volumes reached ~ 150 mm^3^ (18 days), weekly IP treatments of human IgG1 or LY3300054 at 10 mg/kg began for a total of four doses.

### Immune phenotyping of peripheral blood from tumor-bearing mice in humanized models

Peripheral human immune cell engraftment and phenotype was assessed using Trucount™ tubes according to manufacturer’s instruction (BD Biosciences, San Jose, CA). Briefly, 50 μl of blood from hHSC-transplanted mice (day 18, pre-treatment; day 34, after three treatment doses; day 46, after four treatment doses), was added to the tubes and stained with antibodies against human CD45-FITC (BD Biosciences), human CD3-BV786 (Biolegend), human CD4-BV650 (BD Biosciences), human CD8-BV605 (Biolegend, San Diego, CA), and human PD-1-PEeFluor610 (eBiosciences, San Diego, CA) cell surface markers. Samples were subsequently fixed and collected on a 5-laser Fortessa X-20 cytometer (BD Biosciences) and analyzed with FlowJo V10 software (TreeStar). Briefly, approximately 5000 fluorescent beads were collected and enumerated. Human CD45^+^ cells were also gated and enumerated, followed by subsequent gating on CD3^+^cells, followed by CD4^+^ cell and CD8^+^ cell gating and enumeration, and finally PD-1^+^ expressing cells were identified using appropriate IgG control. The absolute number of T cells and CD4+ and CD8+ subsets were calculated based on relative beads collected compared to total number provide by manufacturer. Statistical analysis for human T cell engraftment and phenotype was performed using a two-way ANOVA on repeated measurements.

### Gene expression analysis of tumor and peripheral tissues in humanized tumor models

Total RNA was isolated from snap-frozen tumor tissue (day 15 from H292 model and day 15 post T cell infusion from HCC827 tumor model) or from snap frozen white blood cell pellets, spleens, or bone marrow (hHSC-engrafted models), using the MagMAX 96 Total RNA isolation (Life Technologies, Carlsbad, CA) and RNeasy mini (Qiagen, Hilden, Germany) kits, respectively.

For QuantiGene Plex analysis, 500 ng of total RNA from tumor tissues were subjected to a custom-designed multiplex assay (targets are listed in Additional file 2) according to manufacturer (Affymetrix, Santa Clara, CA) protocol. For nCounter analysis, 100 ng of total RNA from white blood cells were analyzed with the Human Immunology v2 (targets are listed in Additional file 2) nCounter codeset following manufacturer recommendations (NanoString Technologies, Seattle, WA). One- or two-way ANOVA was used for statistical analysis.

## Results

### Binding and blocking properties of LY3300054

**Fig. 1:**
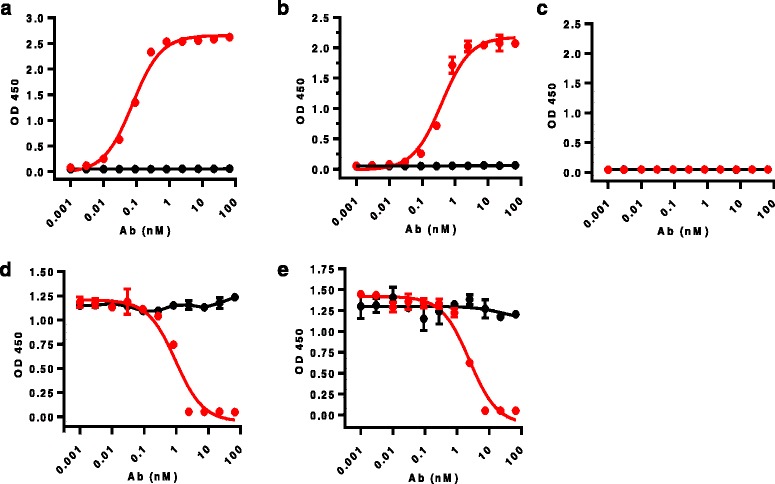
Binding and blocking properties of LY3300054. Panels **a-c**: 96-well plates were coated with 100 ng/well of recombinant (**a**) human, (**b**) cynomolgus or (**c**) murine PD-L1-Fc fusion protein. Bound LY3300054 was detected using HRP-conjugated anti-human Fab antibody and addition of chromogenic substrate (OD at 450 nm). Each data point is the average of two replicates. Data are representative of multiple independent experiments. Panels **d** and **e**: 96-well plates were coated with 100 ng/well of recombinant PD-1 (**d**) or B7–1 protein (EPlate bound PD-L1 was detected using HRP-conjugated streptavidin and addition of chromogenic substrate (OD at 450 nm). Each data point is the average of two replicates. Data are representative of multiple independent experiments.  Human IgG1  LY3300054

ELISA binding assays were performed to assess the selective binding and blocking properties of LY3300054. While LY3300054 bound to human and cynomolgous PD-L1 with similar affinities (EC50 of 0.075 nM and 0.085 nM, respectively) (Fig. 1a, b), LY3300054 did not bind to murine PD-L1 (Fig. 1c); furthermore, LY3300054 did not bind to other proteins of the immunoglobulin superfamily, such as PD-L2, B7–1, B7–2, PD-1, CD28, TIGIT, TIM3, or VISTA (data not shown).

Biacore-based surface plasmon resonance analysis was performed to evaluate the affinity of LY3300054 for binding to Fc-tagged PD-L1. These analyses revealed an affinity of 8.19 × 10^− 11^ M (k_on_, = 1.40 × 10^6^ M^− 1^ s^− 1^; k_off_ = 1.14 × 10^− 4^ s^− 1^). LY3300054 showed cross-reactivity to cynomolgus PD-L1 with a similar affinity (K_D_ 1.22 × 10^− 10^; k_on_ = 1.51 × 10^6^ M^− 1^ s^− 1^; k_off_ = 1.84 × 10^− 4^ s^− 1^), but not with the murine or rat PD-L1.

To assess ligand blocking properties of LY3300054, solid phase blocking ELISA assays were performed. LY3300054 blocked PD-L1 binding to both PD-1 and CD80 ligands in a concentration-dependent manner, with IC50 of 0.95 nM and 2.4 nM, respectively (Fig. 1d, e).

To evaluate the ability of LY3300054 to bind to PD-L1 physiologically expressed on the surface of cells we performed flow cytometry analyses on the tumor cell lines with known surface PD-L1status. For these studies we employed the NCI-H292, and HCC827 tumor cell lines evaluated in the in-vivo studies described below, as well as the PD-L1-negative muscle rhabdomyosa cell line A204 (ATCCCRL-7900), and stained with either the commercially available anti-PD-L1 antibody M1H1or with alexa647 fluor-conjugated LY3300054; as shown in Additional file 1: Figure S1, the PD-L1 –positive NCI-H292 and HCC827 stained robustly with either M1H1 or LY3300054, with the PD-L1 negative A204 cell line failed to stain with either reagent.. Finally, the OV79 cell line employed for the in-vivo studies also stained positive for PD-L1 (data not shown).

### Position N63 on human PDL-1 is a specificity anchor for LY3300054

**Fig. 2:**
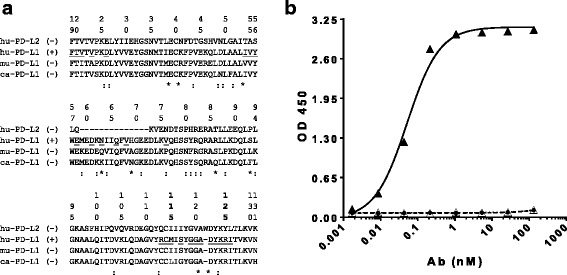
Identification of LY3300054 epitope residues in human PD-L1. Panel **a**: CLUSTALW multiple sequence alignment of domain 1 of human (hu-PD-L1), canine (ca-PD-L1), and murine (mu-PD-L1) PD-L1 and human PD-L2 (huPD-L2) to identify LY3300054 a subset of the specificity anchors on hu-PD-L1. Underlined is the human PD-1 6Ǻ binding site on hu-PD-L1 (according to PDB: 4ZQK (26602187)). An alignment position is marked with (*) if both mu-PD-L1 and ca-PD-L1 substitutions differ from the hu-PD-L1 sequence. An alignment position is marked with (:) if either the mu-PD-L1 or ca-PD-L1 substitution differs from the hu-PD-L1 sequence. Panel **b**: Position N63 on human PD-L1 is a specificity anchor for LY3300054. Canine-to-human mutation K63 N (▲)Wild type ca-PD-L1-Fc (ty, human-to-canine mutant N69H (△). ELISA was repeated twice with three technical replicates for each concentration

Since LY3300054 binds human PD-L1 but not human PD-L2, or murine and canine PD-L1, we performed sequence alignments across each of these proteins to identify key linear residues which might contribute to the specificity of LY3300054 for human PD-L1. The multiple sequence alignment analysis suggested that residues 59–72 of the PD-L1 sequence (59-MEDKNIIQFVHGEE-72) contribute to the human specificity of LY3300054, since this sequence is missing in its entirety from the otherwise homologous sequence of human PD-L2 sequence and is an area of relative divergence across the three tested species (Fig. 2a). In particular, we considered that positions 63 and 69 - side chains, which are exposed to solvent according to PDB: 5C3T [16], might be pivotal for the species specificity of LY3300054 because their corresponding amino acid substitutions diverge among the three PD-L1 sequences (marked with * in the alignment of Fig. 2a). We pursued two mutational strategies to test this hypothesis. The first strategy focused on rescuing the binding of LY3300054 to canine PD-L1 by introducing canine-to-human mutations at positions 63 and 69 of the canine PD-L1-Fc. As shown in Fig. 2b, only the variant K63 N and not N69H rescued the binding of LY3300054 to ca-PD-L1-Fc. Notably, neither mutations (K63 N or N69H) compromise the structural integrity of the ca-PD-L1-Fc protein since the Size Exclusion Chromatography (SEC) profiles of both variants were identical to the SEC profile of the wild type ca-PD-L1-Fc in Additional file 1: Figure S2. The second mutational strategy focused on abrogating the binding of LY3300054 to human PD-L1 by introducing human-to-murine mutations at positions 63 and 69 in human PD-L1. Only N63Q and not H69A abrogated the binding of LY3300054 to hu-PD-L1-Fc Additional file 1: Figure S3. Thus, both mutational strategies demonstrated that the N63 residue is pivotal for the species specificity of LY3300054. Furthermore, analysis of the co-crystal structure of the human PD-1/PD-L1 co-crystal indicates that the N63 residue is part of the 6Ǻ binding site of PD-1 on PD-L1 ([16]). Hence, LY3300054 blocks the PD-1/PD-L1 interaction because its binding epitope overlaps the binding site of PD-1 on PD-L1.

### Functional activity of LY3300054 in vitro

**Fig. 3:**
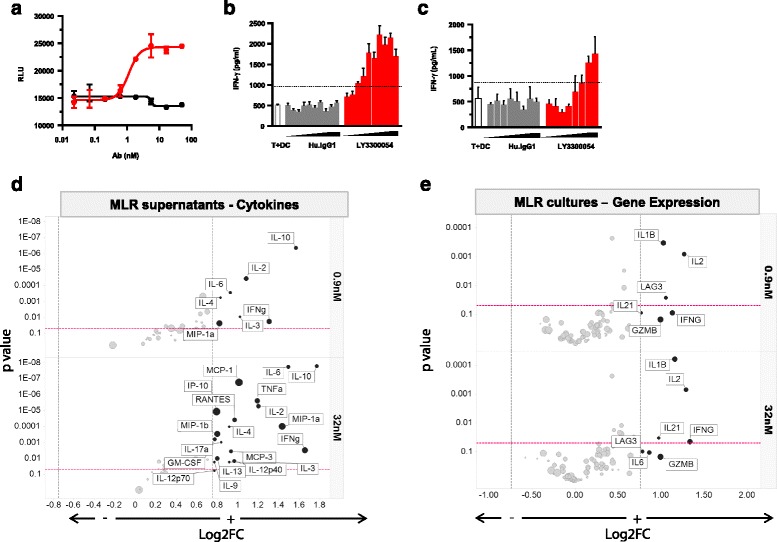
LY3300054 enhances T cell activation in vitro. Panel **a**: Jurkat-NFAT reporter assay: Each data point represents the average of two technical replicates, with error bars representing the SD. Data are representative of three independent experiments. Panel **b**: Mixed leukocyte reactions. Supernatants were measured for IFN-γ production by ELISA. Each data point represents the average of 8 replicates, with error bars representing the SEM. Data are representative of multiple experiments and donor T cells/DC pairs. Panel **c**: Tetanus toxoid recall assay: Supernatants were measured for IFN-γ production by ELISA. Each data point represents the average of 4 replicates, with error bars representing the SD. Data are representative of two experiments with PBMC obtained from different donors. Panels **d** and **e**: Gene expression analysis of cell lysate (E) and cytokine level analysis of cell culture supernatant (F) from the mixed leukocyte reactions using QuantiGene Plex and microbead-based immunoassay panels, respectively. Volcano plots show Log2 fold change of gene expression (E) or cytokine levels (F) in the LY3300054 treated group compared to control group. The highlighted circles correspond to differentially expressed genes (DEG) or cytokines that display fold change > 1.7 (black solid vertical line) and *p* value < 0.05 (horizontal dotted line). Circle sizes are proportional to the level of expression in LY3300054 group. One-way ANOVA was used for statistical analysis.  Human IgG1  LY3300054

The ability of LY3300054 to enhance T cell functional activity was assessed using a variety of in vitro assays. In a PD-1 reporter assay, using Jurkat cells engineered to stably express human PD-1 and an NFAT-luciferase reporter construct and CHO-K1 cells engineered to stably express human PD-L1, addition of LY3300054 resulted in a concentration-dependent increase in NFAT-driven expression of luciferase, overcoming the inhibitory effects of PD-L1 expressed by CHO cells (Fig. 3a). In mixed leukocyte reactions (MLR) using allogeneic human DC and T cells, addition of LY3300054 enhanced the allogeneic T cell response in a dose-dependent manner, with activity observed at concentrations as low as 0.05 nM as measured by IFN-γ secretion and mRNA expression (Fig. 3b, e). Additional analysis of MLR cultures by 41-plex microbead-based cytokine and gene expression panels revealed enhanced secretion and transcription of multiple immune factors in response to LY3300054 treatment exemplified by increased levels of IL-6, TNFα, MCP-1, MIP-1α, MIP-1β, RANTES, IP-10, IL-4, IL-13, IL-12 in culture medium (Fig. 3d) and upregulation of *IL2, IL1B, IL21* genes (Fig. 3e). Finally, LY3300054 was also shown to enhance T cell activity in the tetanus-toxoid recall assay (TTRA) which measures the ability to stimulate antigen-specific memory T cells in PBMC. (Fig. 3c).

**Fig. 4:**
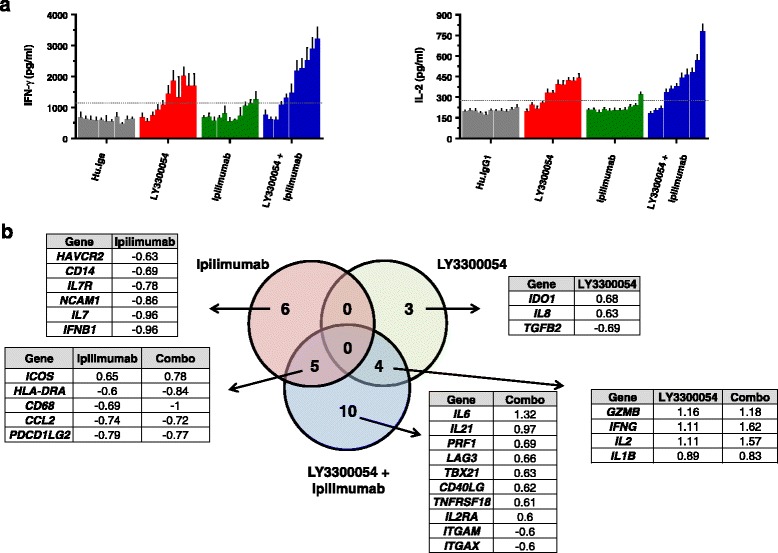
Combination of LY3300054 and ipilimumab enhances T cell activation in vitro. Panel **a**: Mixed leukocyte reactions. Allogeneic DC were co-cultured with purified CD4^+^ T cells for 72 h in the presence of increasing (two-fold increments) concentrations of LY3300054, ipilimumab or a combination of both antibodies ranging from 0.0003 to 67 nM. Supernatants were measured for IFN-γ and IL-2 production by ELISA. Each data point represents the average of 8 replicates, with error bars representing the SEM. Data were generated with four different PBMC donors. Panel **b**: Gene expression analysis of cell lysate from the mixed leukocyte reactions was performed using QuantiGene Plex assay. Venn diagram showing the number of shared (overlap circle) and treatment-specific (no overlap) DEGs across the different treatments. Tables list the Log2 fold-change of LY3300054 vs control group for genes with fold-change > 1.5, *p* value < 0.05. One-way ANOVA was used for statistical analysis

In MLR assays, LY3300054 also displayed biological activity in combination with the anti-CTLA4 antibody ipilimumab. In these experiments, which utilized equimolar concentrations of LY3300054 and ipilimumab, IFNγ and IL2 secretion was substantially enhanced in the combination treatment compared to each of the single agents (Fig. 4a). High-content gene expression analysis revealed overlapping gene expression changes across all treatment groups, and also, in agreement with previous reports on the combination of PD-1 and ipilumimab therapy ([29]), distinct gene profiles in the combination group, with the maximum treatment effect observed at 67.5 nM (Fig. 4b). LY3300054 single agent treatment induced gene expression changes indicative of immune activation, exemplified by increased expression of *IFNG, IL2, IDO1, GZMB, IL1B, IL6*, while ipilimumab single agent treatment resulted in enhanced T cell activation, exemplified by enhanced *ICOS, and IFNG* accompanied by downregulation of myeloid genes (*CD68, CD14, HLA-DRA*). The combination of LY3300054 and ipilimumab further upregulated T-cell specific genes reflecting a Th1 response (*IFNG, IL2, TBX21*), T-cell activation (*IL2, IFNG, ICOS*) and downregulation of myeloid genes (*CD68, CD14, HLA-DRA*).

### LY3300054 ADCC and CDC functions

LY3300054 was engineered to ablate Fc-gamma receptor engagement and associated immune effector functions, specifically ADCC and CDC. LY3300054 was evaluated by SPR and solid phase ELISA to lack binding to FcgRI, FcgRIIa, and FcgRIIIa (F158) within the limit of detection titrated to 10 μM antibody concentration (data not shown). Ablation of ADCC and CDC functions of LY3300054 was evaluated in cell-based assays, using the HEL PD-L1 positive tumor cell lines. In both ADCC and CDC assays LY3300054 did not direct detectable effector function activity against HEL target cells, while rituximab was shown to mediate significant ADCC and CDC response against CD20-positive Wil2-S cells (Additional file 1: Figure S4A, B).

### LY3300054 does not trigger non-specific cytokine production by PBMC

We evaluated the ability of LY3300054 to result in non-specific cytokine release from unstimulated human PBMC using plate-bound cytokine release assays. While incubation of PBMC with anti-CD3ε or the anti-CD28 agonist antibody TGN1412 antibodies resulted in robust cytokine production for a number of cytokines, including those associated with cytokine release syndrome (CRS) (TNFα, IL-6, IL-2, IFNγ, and IL-1β), incubation of donor PBMC with LY3300054 did not result in significant levels of cytokine release for any of the evaluated cytokines over a broad range of concentrations from 0.003 to100 μg/ml (Additional file 1: Figure S5).

### Biological activity of LY3300054 in humanized murine models in vivo

We evaluated the functional activity of LY3300053 in vivo using human tumor xenograft models and immune deficient NSG animals reconstituted with human immune cells. We performed these studies in different models, including a preventative (co-implantation) model, a therapeutic model with established tumors and animals reconstituted with allogeneic human T cells, and two therapeutic models with established tumors and animals engrafted with human sHSC), each designed to evaluate different functional attributes of anti-PD-L1 immunotherapy. Each of these models evaluates the ability to modulate the inherent alloreactivity of the engrafted human immune system against the tumor. In each model, we evaluated the effects of LY3300054 on anti-tumor alloreactivity, and also performed detailed intra-tumoral and peripheral immune pharmacodynamic assessments to evaluate how LY3300054 therapy modulated human immune cell activities. Notably, each of the tumor cell lines evaluated in these studies robustly expresses HLA class I (Additional file 1: Figure S6).

**Fig. 5:**
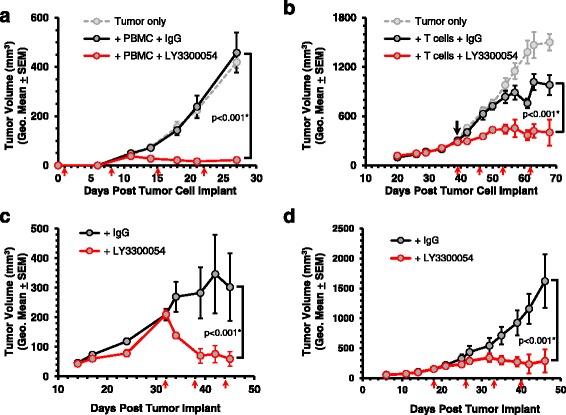
LY3300054 demonstrates anti-tumor efficacy in xenograft tumor models reconstituted with human immune cells. Antibody treatment (indicated by red arrows), either human IgG or LY3300054, was delivered by intra-peritoneal injection in each case at 10 mg/kg. Tumor growth was monitored by caliper, and results are represented as a geometric mean of tumor volumes ± SEM. Panel **a**: Co-implantation model: NCI-H292 tumor cells and freshly isolated human PBMC were co-implanted subcutaneously into the flanks of NSG mice. *n* = 8 for all groups. Panel **b**: Established tumor model: HCC827 tumor cells were implanted subcutaneously into the flanks of NSG mice. When tumors reached volumes of ~ 300 mm^3^ (approximately 5 weeks), mice were infused with previously expanded human T cells (black arrow). *n* = 8 for all groups. Panels **c** and **d**: Established tumor models in CD34+ hHSC-reconstituted animals: HCC827 tumors (NSG, panel |C) or OV79 tumors (NOG, panel D). Mice were implanted subcutaneously with either HCC827 or OV79 tumor fragments at ~ 15–17 weeks of age (~ 13–15 weeks post HSC engraftment). HCC827 tumors were allowed to grow to ~ 200 mm^3^ (4 weeks) and OV79 were allowed to grow to ~ 150 mm^3^ (18 days) before starting weekly treatments of either human IgG1 or LY3300054 at 10 mg/kg. *n* = 5–9 per group. Statistically significant difference is indicated* (two-way repeated measurement ANOVA, RM-ANOVA)

For the preventative studies, mice were co-implanted with a mixture of human PBMC and NCI-H292 tumor cells, followed by treatment with LY3300054 or control human IgG1. Compared to untreated and human IgG1-treated animals, treatment with LY3300054 resulted in significant tumor inhibition (*p* < 0.001) (Fig. 5a). The therapeutic potential of LY3300054 to modulate T cell-mediated anti-tumor activity in an established tumor setting was evaluated using the HCC827 xenograft mouse tumor model, and animals reconstituted with ex-vivo expanded CD3+ T cells. While infusion of expanded human T cells alone modestly delayed tumor growth, reflecting the baseline anti-tumor reactivity of the engrafted alloreactive T cells, treatment with LY3300054 significantly enhanced this effect resulting in potent anti-tumor activity (Fig. 5b). The therapeutic potential and activity of LY3300054 in the context of more completely human immune-replete animals was assessed in immunodeficient NSG or NOG mice engrafted with HSCs of human origin (CD34+ huHSCs), and two xenograft mouse tumor models, using the HCC827 and OV79 tumor cell lines. These experimental models display a more replete human immune compartment exemplified by differentiation of both lymphoid and myeloid cells ([30]). In both models LY3300054 therapy strongly enhanced the alloreactive anti-tumor response (Fig. 5c-d).

**Fig. 6:**
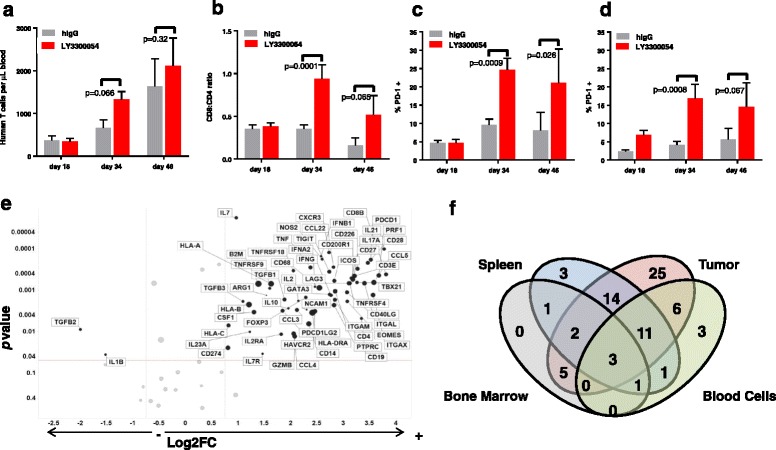
LY3300054 enhances peripheral T cell engraftment and activation and induces T cell inflamed phenotype in tumor tissues of CD34+ hHSC-engrafted NOG and NSG mice. Panels **a**, **b**, **c**, **d**: Blood from OV79-bearing CD34+ hHSC-engrafted NOG mice was analyzed for human T cell engraftment and phenotype using TruCount tubes on day 18 (pre-dose), day 34 (after three antibody doses), and day 46 (after four antibody doses). Peripheral T cell engraftment (A); CD8:CD4 ratio (B); PD-1 expression in CD4+ (C) and CD8+ T cells (D) cells. Results are represented as a geometric mean of engraftment + SEM with *n* = 9 mice on day 18 and day 34, and *n* = 5 mice on day 46. Two-way repeated measurements ANOVA was used for statistical analysis. Panel **e**: Gene expression analysis of tumor sample was performed using QuantiGene Plex assay. Volcano plots show Log2 fold-change of gene expression in the LY3300054 treated group compared to control group. The highlighted circles correspond to differentially expressed genes that display fold change > 1.7 (black solid vertical line) and *p* value < 0.05 (horizontal dotted line). Circle sizes are proportional to the level of expression in LY3300054 group. One-way ANOVA was used for statistical analysis. Panel **f**: Venn diagram showing the number of shared and tissue-specific DEGs (LY3300054 vs human IgG treatment) across various tissue types. Table on the right lists shared DEGsacross various tissues with fold-change > 1.7, *p* value < 0.05 for LY3300054 vs control

To evaluate the intra-tumoral and peripheral immune-pharmacodynamic effects of LY3300054 therapy on engrafted human immune cells, tissues (tumor, spleen, bone marrow, peripheral blood) were collected from the above models and subjected to mechanistic analyses. In CD34+ hHSC-reconstituted, tumor-bearing models, treatment with LY3300054 resulted in an increase of the absolute number of human T cells, an enhanced CD8/CD4 T cell ratio, and increased frequency of PD1+ CD8+ and CD4+ T cells, indicative of T cell activation (Fig. 6a-d). Furthermore, LY3300054 therapy resulted in prominent immune-related gene expression changes consistent with IFNγ pathway and T cell activation in the tumor tissue (Fig. 6e), as well as in spleen (Additional file 1: Figure S7A) and peripheral blood cells (Additional file 1: Figure S7B), and to a much lesser extent in the bone marrow (Additional file 1: Figure S7C). In tumor tissue of CD34+ hHSC-reconstituted mice, LY3300054-induced increased gene expression changes reflected T cell infiltration and activation (*CD3E, CD8B, CD4, PRF1, GZMB*, *TBX21, EOMES*), myeloid cell infiltration and differentiation (*ITGAM, ITGAX, CD14, CD68, ARG1*) upregulation of co-inhibitory/co-stimulatory receptors and ligands (*TNFRSF9, TNFRS18, TNFRSF4, CD28, CD27, ICOS, CD226, CD200R1, PDCD1, CD274, PDCD1LG2, TIGIT, HAVCR2, LAG3*), cytokines and their receptors (*IFNG, IL2, IL2RA, IL21, CCL3, CCL4, CCL5*), interferon type I response (*IFNA2, IFNB1*), antigen presentation and MHC class I and II (*HLA-A, HLA-B, HLA-C, B2M, HLA-DRA*), and down-regulation of only two genes from the tested panel, *TGFB2* and *IL1B* (Fig. 6e). Three genes indicative of T cell activation *CD274* (PD-L1)*, CCL5* (RANTES), and *ITGAL* (LFA-1) were upregulated in all 4 tissues evaluated, demonstrating a systemic effect of PD-L1 blockade in HCC827-bearing CD34+ HSC-reconstituted NOG mice. While the gene profiles between tumor, spleen, and peripheral blood (the three tissues with robust gene modulation) showed patterns of overlap, unique patterns of expression were also apparent, suggesting tissue- and tumor-specific effects of LY3300054 (Fig. 6f). Furthermore, LY3300054 also upregulated genes indicative of T cell activation (upregulated *CD274, PDCD1LG2, IDO1, CXCL9, CXCL10, CD3E, CD8B, CD4, ICOS, CD27* etc) in tumor tissues collected from the established HCC827 tumor model implanted with ex vivo expanded human T cells and the co-implantation NCI-H292 model, although the overall effect of the antibody was less pronounced in these models compared to HCC827-bearing CD34+ hHSC-reconstituted NSG mice (Additional file 1: Figure S8).

## Discussion

We describe the discovery and pre-clinical characterization of LY3300054, an Fc effector function-silenced fully human antibody that binds to the PD-L1 checkpoint ligand and blocks interactions with both PD-1 and CD80 receptors. LY3300054 is shown to lack ADCC and CDC effector functions, and to not trigger TCR-independent T cell activation as assessed by non-specific cytokine production by human PBMC in vitro. From a structural perspective, using sequence analysis and mutagenesis we identify a key residue on PD-L1, N63, which is part of the binding site for PD-1, may play important roles in the function of the PD-1/PD-L1 interaction, and which contributes to the species specificity of LY3300054. In vitro LY3300054 is shown to block PD-L1-mediated T cell suppression of primary human T cells in both primary MLR and tetanus-toxoid recall assays, and to reverse TCR-engagement mediated activation of the NFAT pathway using signal reporter Jurkat cells with ectopic expression of human PD-1. In both preventative and therapeutic xenograft tumor models reconstituted with human T cells or HSC, LY3300054 therapy resulted in robust anti-tumor activity, accompanied by the development of distinct T cell inflamed signatures in the tumor and peripheral tissues.

Although multiple agents that block the PD-1/PD-L1 axis have been described and evaluated in the clinic, relatively little information exists about the functional properties of these molecules in the pre-clinical in vivo setting. The paucity of information is related at least in part to the relatively recent availability of humanized murine tumor models with reconstituted human immune cell compartment(s). We chose to thoroughly evaluate the functional activity of LY3300054 in a variety of immune-humanized mouse models, to begin to understand how anti-PD-L1 therapy might modulate anti-tumor T cell immunity in biologically complex and relevant matrices, more representative of the clinical setting. These models represented both prophylactic and therapeutic intervention, reconstituted with human PBMC, expanded human T cells, or CD34^+^ HSC. In each of the tested models, LY3300054 treatment resulted in enhanced anti-tumor alloreactivity and robust anti-tumor effects, demonstrating the potency of the agent as well as the relevance of the PD-1/PD-L1 axis in the context of humanized mouse models.

We took advantage of the ability to collect tumor and tissues from the animal models and performed high content flow cytometry and molecular immuno-pharmacodynamic profiling to obtain insights about the mechanism of action of LY3300054-mediated anti-PD-L1 blockade in each of the humanized models. These analyses clearly demonstrated the ability of LY3300054 to modulate human T cell functions as reflected by increased peripheral T cell numbers and in particular CD8^+^ T cells, and enhanced activation status of peripheral allo- and/or xeno-reactive T cells triggered through TCR engagement. High-content gene expression profiling revealed that LY3300054-induced a T cell inflamed phenotype in tumor tissues across all models tested, and almost exclusively resulted in upregulation of gene expression; these data demonstrate the ability of LY3300054 to effectively block the PD-L1/PD-1 axis and to activate T cells to drive more effective anti-tumor T cell immunity. Within a model, while a core set of differentially expressed genes were shared across tumor and normal tissues including spleen, peripheral blood and bone marrow, a considerable number of genes were upregulated in a tissue-specific manner suggesting that LY3300054 activity might be context-dependent.

In more immune-replete animals, blockade of the PD-L1/PD-1 axis activated additional pathways beyond T lymphocytes, including genes associated with adaptive immunity such as co-stimulatory and co-inhibitory receptors, cytokines and transcription factors, and innate immune pathways such as interferon, MHC, and myeloid pathways. Whether these observations reflect a direct effect of PD-L1 blockade on innate immune cells or an indirect effect resulting from T cell activation, they underscore the diverse and to-date not fully appreciated role for PD-L1 blockade to modulate the activity and function of cell types beyond T lymphocytes, and highlight the need to both integrate and evaluate innate immune modulation and cell subsets in the context of anti-PD-L1-based immunotherapies.

## Conclusion

LY3300054 is currently in phase I clinical studies to evaluate activity as monotherapy and in combination with other agents.

## Additional files

Additional file 1: **Figure S1.** PD-L1 expression on tumor cell lines. **Figure S2.** SEC profiles of dog PD-L1-Fc and its dog-to-human variants K63 N and N69H. **Figure S3.** Position N63 on human PD-L1 is a specificity anchor for LY3300054. **Figure S4.** LY3300054 does not induce ADCC or CDC in vitro. **Figure S5.** LY3300054 does not induce non-specific cytokine production by human PBMCs in vitro. **Figure S6.** HLA Class I expression profile on tumor cell lines. **Figure S7**. LY3300054 enhances expression of immune-related genes in peripheral tissue of CD34+ HSC transplanted mice bearing OV79 tumors (NOG). **Figure S8** LY3300054 enhances expression of immune-related genes in NCI-H292 and HCC827 tumors from humanized mouse tumor models. (PPTX 1085 kb)

Additional file 2: Supplementary Methods. (DOCX 34 kb)
