# Correction to: Discovery and preclinical characterization of the antagonist anti-PD-L1 monoclonal antibody LY3300054

**DOI:** 10.1186/s40425-018-0354-6

**Published:** 2018-06-04

**Authors:** Yiwen Li, Carmine Carpenito, George Wang, David Surguladze, Amelie Forest, Maria Malabunga, Mary Murphy, Yiwei Zhang, Andreas Sonyi, Darin Chin, Douglas Burtrum, Ivan Inigo, Anthony Pennello, Leyi Shen, Laurent Malherbe, Xinlei Chen, Gerald Hall, Jaafar N. Haidar, Dale L. Ludwig, Ruslan D. Novosiadly, Michael Kalos

**Affiliations:** 1 Lilly Research Laboratories, Department of Cancer Immunobiology, New York, NY USA; 2 Lilly Research Laboratories, Department of Preclinical Pharmacology, New York, NY USA; 3 Lilly Research Laboratories, Department of Biologics Technology, New York, NY USA; 4 Lilly Research Laboratories, Department of Non-Clinical Safety, Indianapolis, IN USA; 5 Lilly Research Laboratories, Department of Quantitative Biology, New York, NY USA; 6 Eli Lilly and Company, 450 East 29th Street, New York, NY 10016 USA; 7 Janssen Pharmaceutical Companies of Johnson and Johnson, Springhouse, PA USA

## Correction

Unfortunately, after publication of this article [1], it was noticed that corrections to the legends of Figs. 1 and 2 were not correctly incorporated. The correct legends can be seen below.

The original article has also been updated.

**Figure 1** Binding and blocking properties of LY3300054. Panels **a–c**: 96-well plates were coated with recombinant human (**a**), cynomolgus (**b**), or murine (**c**) PD-L1-Fc fusion protein (100 ng/well each). Bound LY3300054 was detected using HRP-conjugated anti-human Fab antibody and addition of chromogenic substrate (OD at 450 nm). 96-well plates were coated with 100 ng/well of recombinant PD-1 (**d**) or B7-1 protein (**e**), then incubated with a mixture of biotin-conjugated PD-L1 and either LY3300054 or human IgG1 antibodies. Plate bound PD-L1 was detected using HRP-conjugated streptavidin and addition of chromogenic substrate (OD at 450 nm). In all experiments, each data point is the average of two replicates. Data (**a-e**) are representative of multiple independent experiments

**Figure 2** Identification of LY3300054 epitope residues in human PD-L1. Panel **a**: CLUSTALW multiple sequence alignment of domain 1 of human (hu), canine (ca), and murine (mu) PD-L1 and hu-PD-L2 to identify the LY3300054 species specificity anchors on hu-PD-L1. Underlined is the human PD-1 6 Å binding site on hu-PD-L1 (according to PDB: 4ZQK (26602187)). An alignment position is marked with (*) if both mu-PD-L1 and ca-PD-L1 substitutions differ from the hu-PD-L1 sequence. An alignment position is marked with (:) if either the mu-PD-L1 or ca-PD-L1 substitution differs from the hu-PD-L1 sequence. Panel **b**: Position N63 on human PD-L1 is a specificity anchor for LY3300054. Canine-to-human mutation K63 N (▲) rescues the ELISA binding of LY3300054 to canine PD-L1. Like wild type ca-PD-L1-Fc (●), canine-to-human mutant N69H (△) does not bind LY3300054
