# Supplementary material for: Discovery and preclinical characterization of the antagonist anti-PD-L1 monoclonal antibody LY3300054
Source: J Immunother Cancer. 2018 Apr 30;6:31. doi: 10.1186/s40425-018-0329-7 (PMC5925824; doi:10.1186/s40425-018-0329-7)
Supplement: Supplementary file 1 — Figure S1. PD-L1 expression on tumor cell lines. Figure S2. SEC profiles of dog PD-L1-Fc and its dog-to-human variants K63 N and N69H. Figure S3. Position N63 on human PD-L1 is a specificity anchor for LY3300054. Figure S4. LY3300054 does not induce ADCC or CDC in vitro. Figure S5. LY3300054 does not induce non-specific cytokine production by human PBMCs in vitro. Figure S6. HLA Class I expression profile on tumor cell lines. Figure S7. LY3300054 enhances expression of immune-related genes in peripheral tissue of CD34+ HSC transplanted mice bearing OV79 tumors (NOG). Figure S8 LY3300054 enhances expression of immune-related genes in NCI-H292 and HCC827 tumors from humanized mouse tumor models. (PPTX 1085 kb) [file 40425_2018_329_MOESM1_ESM.pptx]

## Slide 1
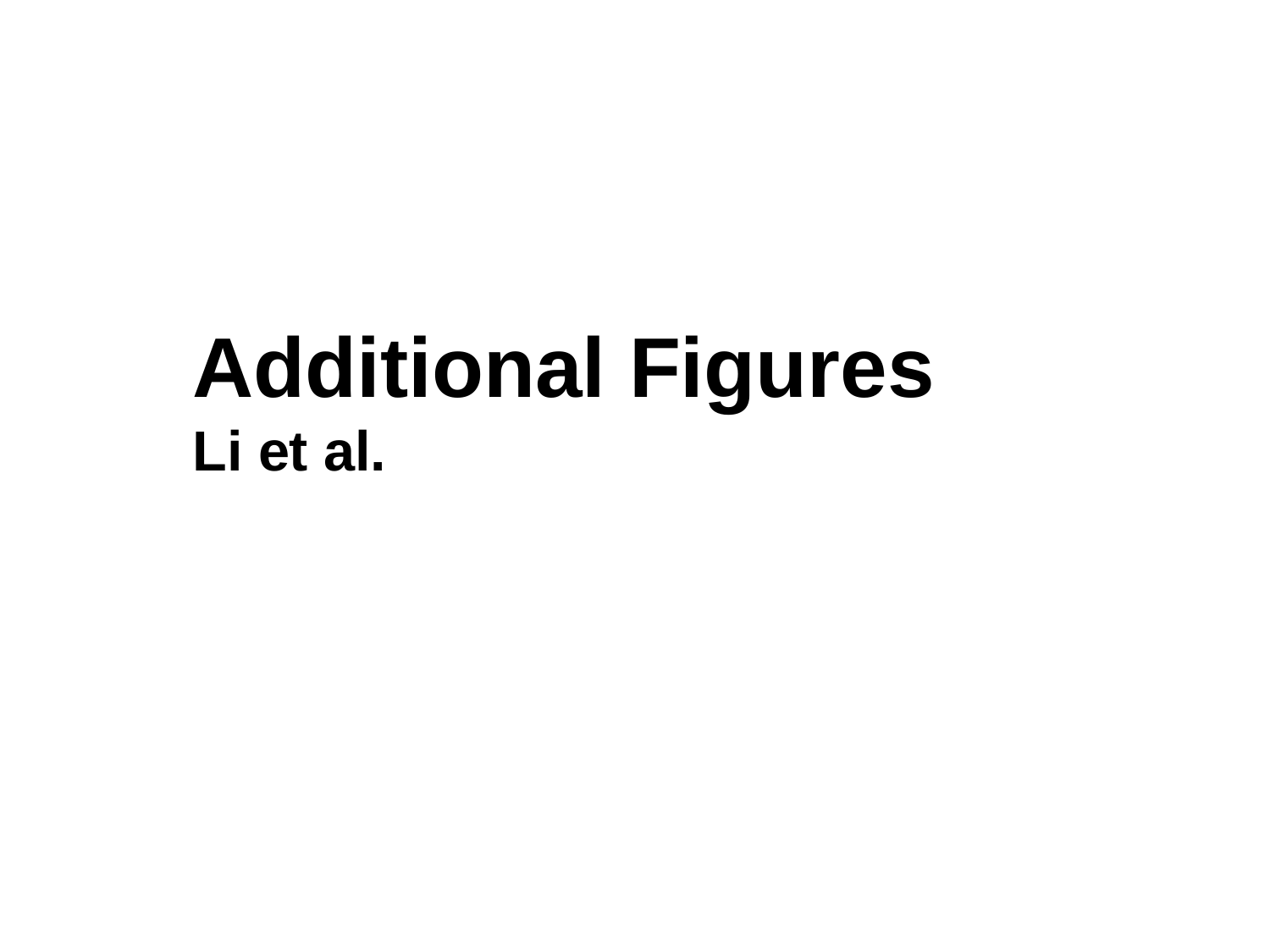

Additional Figures
Li et al.

## Slide 2
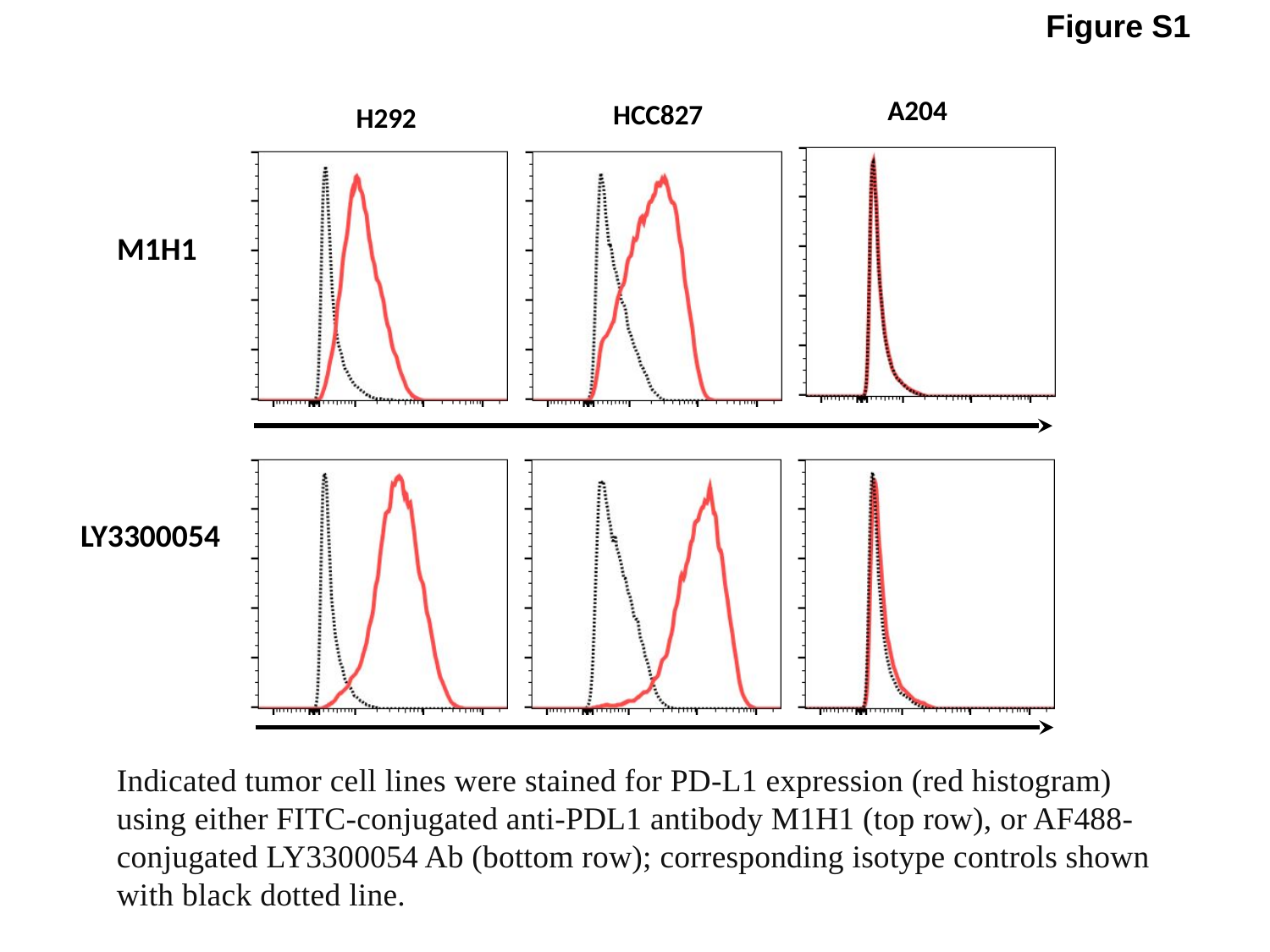

Figure S1
A204
HCC827
H292
M1H1
LY3300054
Indicated tumor cell lines were stained for PD-L1 expression (red histogram) using either FITC-conjugated anti-PDL1 antibody M1H1 (top row), or AF488-conjugated LY3300054 Ab (bottom row); corresponding isotype controls shown with black dotted line.

## Slide 3
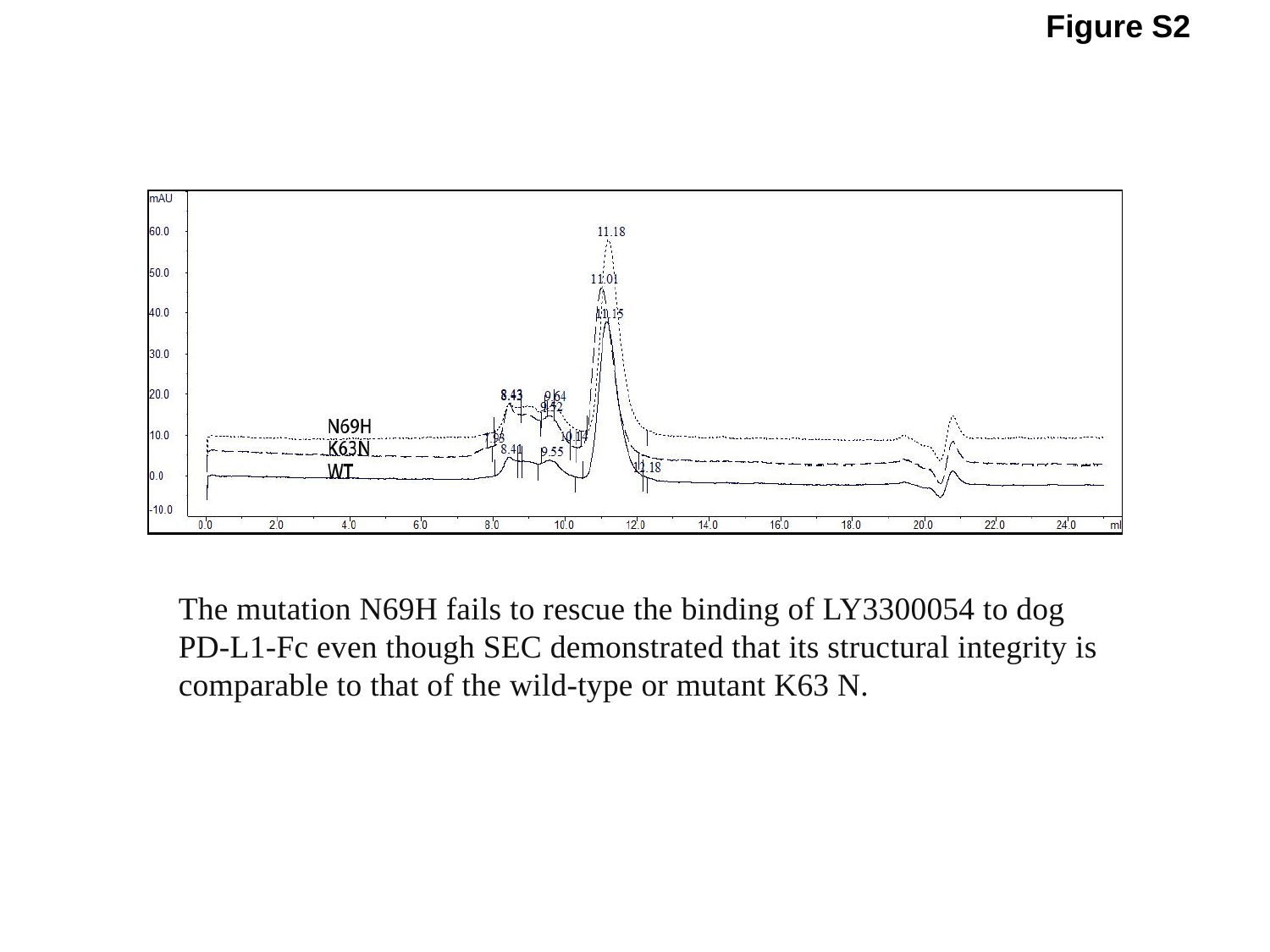

Figure S2
The mutation N69H fails to rescue the binding of LY3300054 to dog PD-L1-Fc even though SEC demonstrated that its structural integrity is comparable to that of the wild-type or mutant K63 N.

## Slide 4
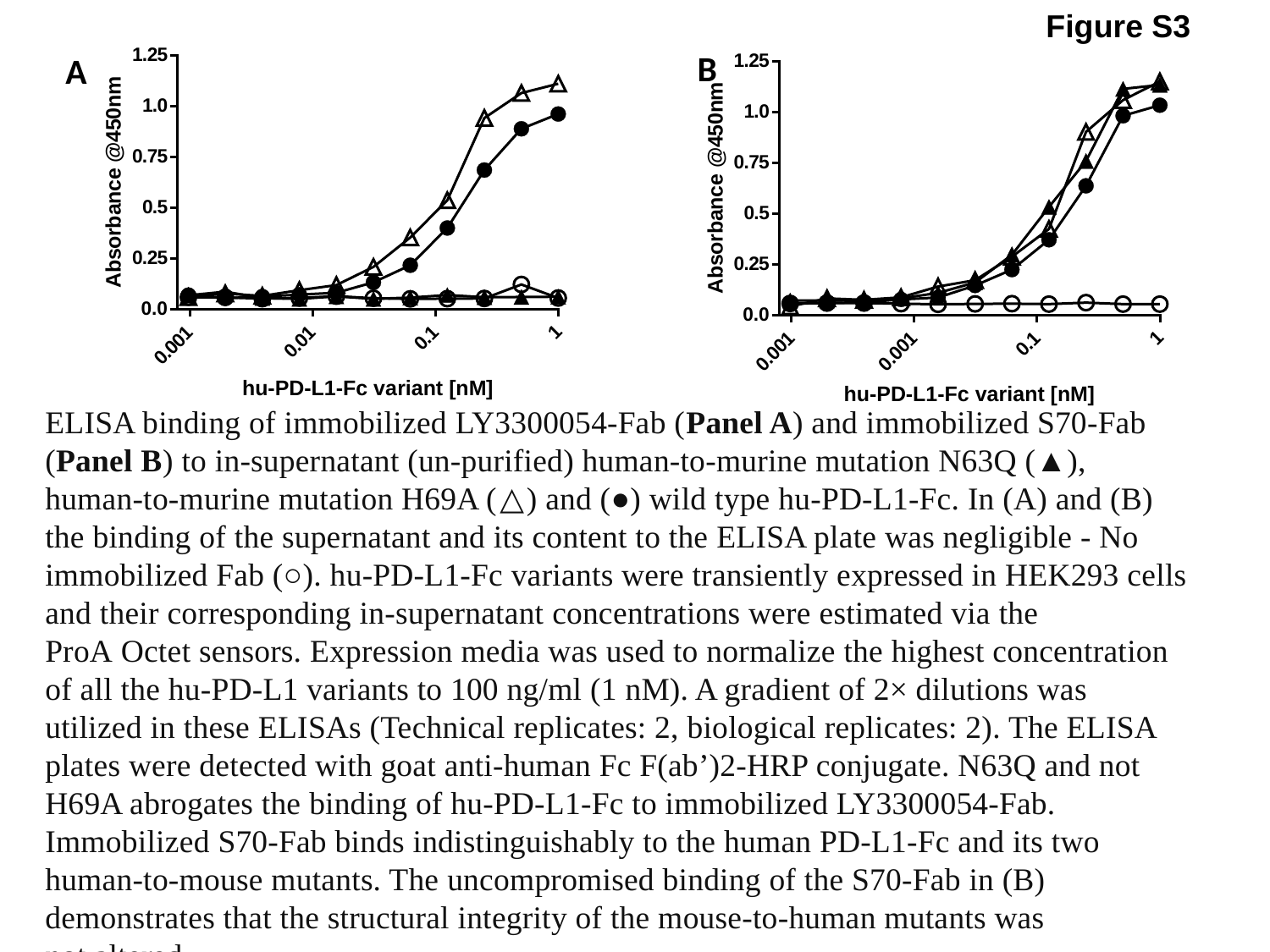

Figure S3
B
A
ELISA binding of immobilized LY3300054-Fab (Panel A) and immobilized S70-Fab (Panel B) to in-supernatant (un-purified) human-to-murine mutation N63Q (▲), human-to-murine mutation H69A (△) and (●) wild type hu-PD-L1-Fc. In (A) and (B) the binding of the supernatant and its content to the ELISA plate was negligible - No immobilized Fab (○). hu-PD-L1-Fc variants were transiently expressed in HEK293 cells and their corresponding in-supernatant concentrations were estimated via the ProA Octet sensors. Expression media was used to normalize the highest concentration of all the hu-PD-L1 variants to 100 ng/ml (1 nM). A gradient of 2× dilutions was utilized in these ELISAs (Technical replicates: 2, biological replicates: 2). The ELISA plates were detected with goat anti-human Fc F(ab’)2-HRP conjugate. N63Q and not H69A abrogates the binding of hu-PD-L1-Fc to immobilized LY3300054-Fab. Immobilized S70-Fab binds indistinguishably to the human PD-L1-Fc and its two human-to-mouse mutants. The uncompromised binding of the S70-Fab in (B) demonstrates that the structural integrity of the mouse-to-human mutants was not altered.

## Slide 5
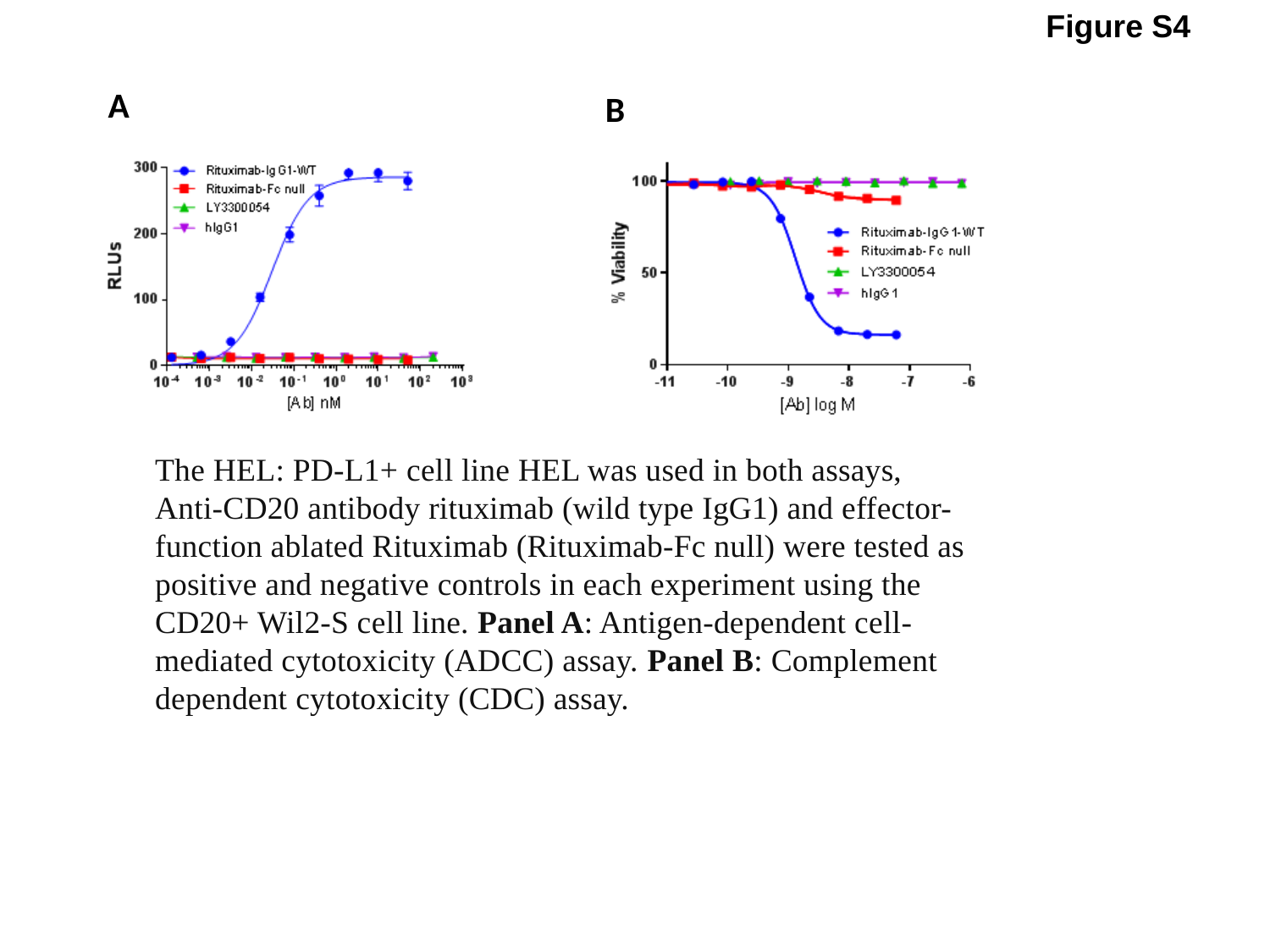

Figure S4
A
B
The HEL: PD-L1+ cell line HEL was used in both assays, Anti-CD20 antibody rituximab (wild type IgG1) and effector-function ablated Rituximab (Rituximab-Fc null) were tested as positive and negative controls in each experiment using the CD20+ Wil2-S cell line. Panel A: Antigen-dependent cell-mediated cytotoxicity (ADCC) assay. Panel B: Complement dependent cytotoxicity (CDC) assay.

## Slide 6
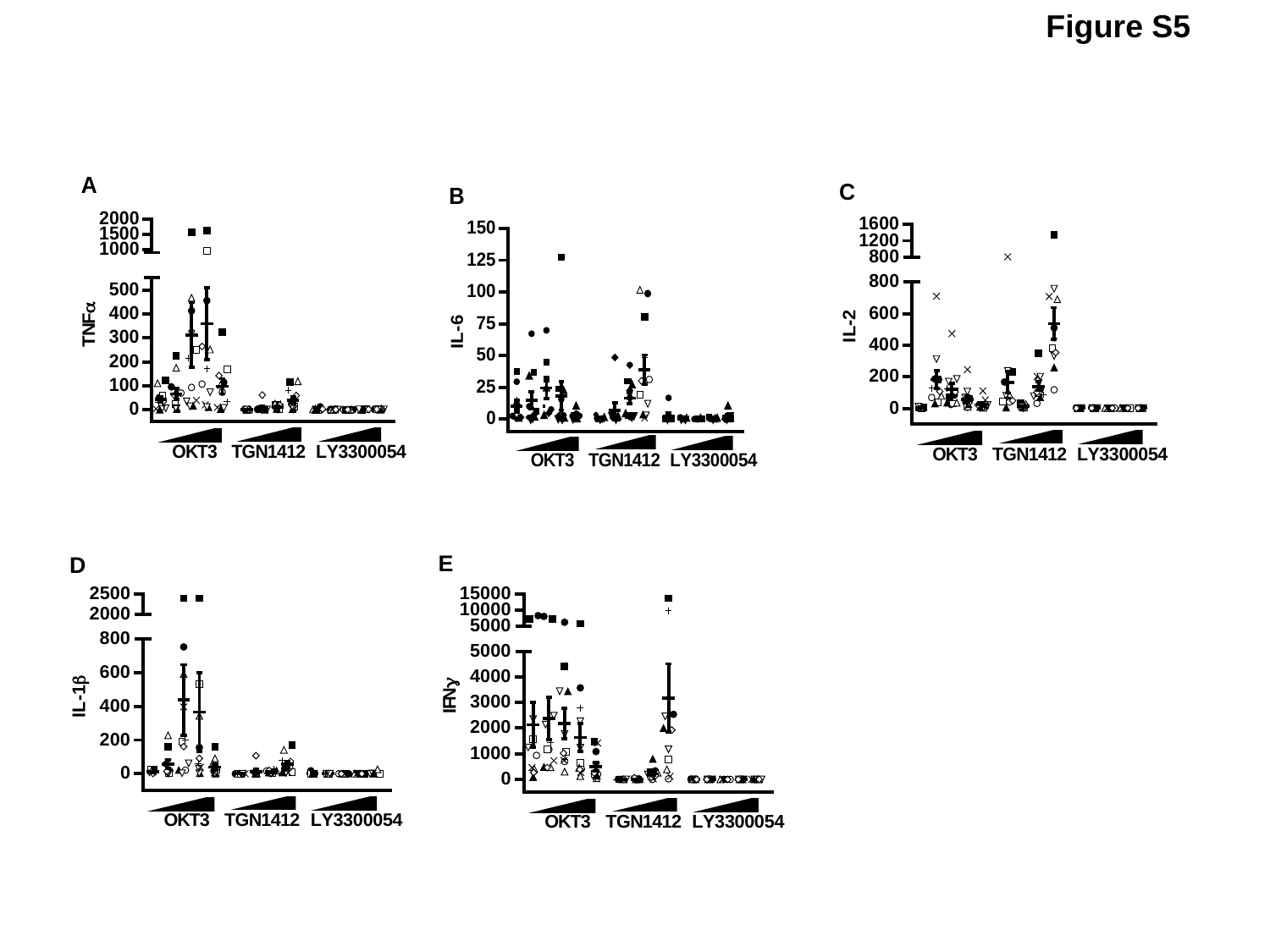

Figure S5

## Slide 7
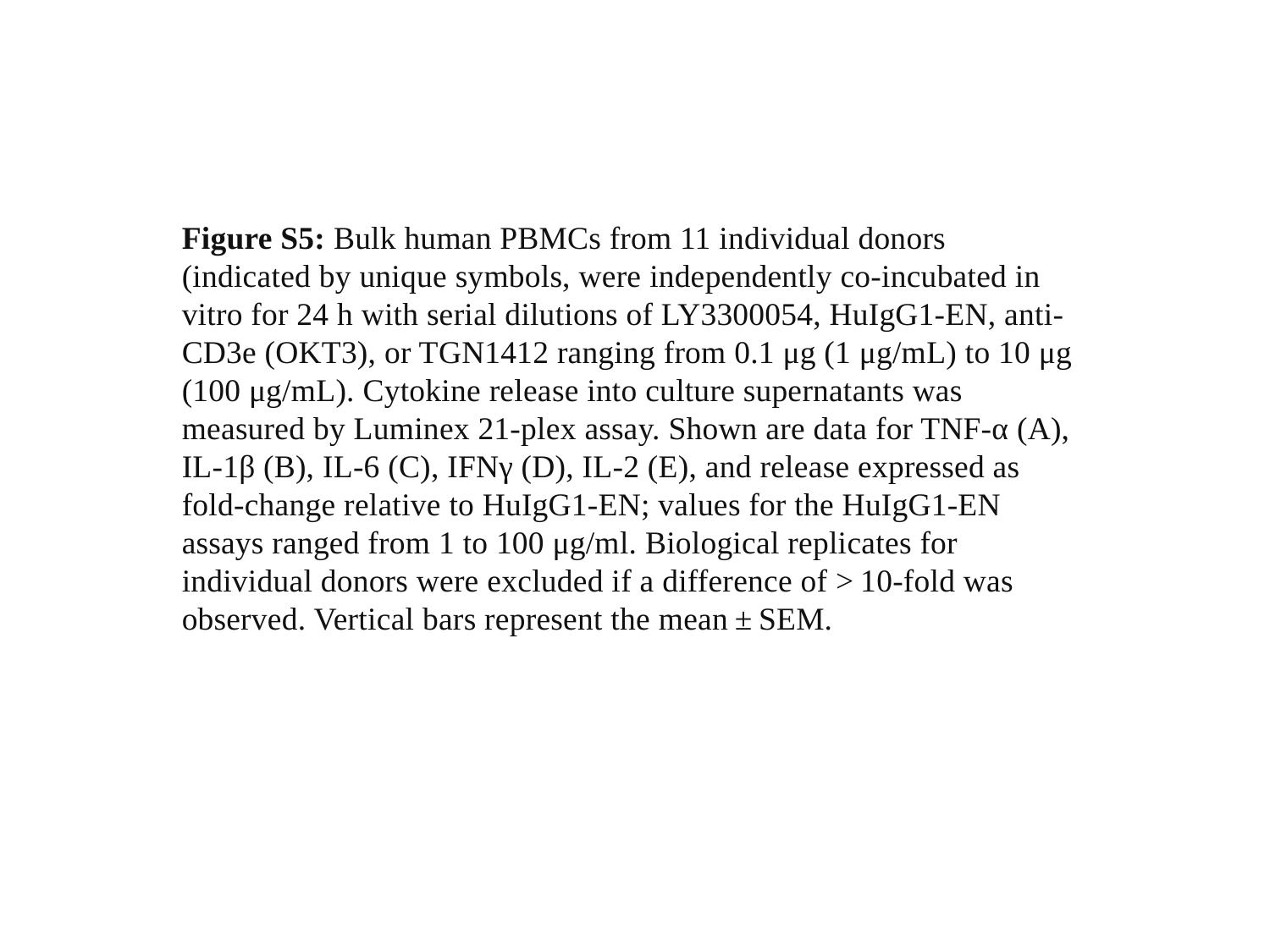

Figure S5: Bulk human PBMCs from 11 individual donors (indicated by unique symbols, were independently co-incubated in vitro for 24 h with serial dilutions of LY3300054, HuIgG1-EN, anti-CD3e (OKT3), or TGN1412 ranging from 0.1 μg (1 μg/mL) to 10 μg (100 μg/mL). Cytokine release into culture supernatants was measured by Luminex 21-plex assay. Shown are data for TNF-α (A), IL-1β (B), IL-6 (C), IFNγ (D), IL-2 (E), and release expressed as fold-change relative to HuIgG1-EN; values for the HuIgG1-EN assays ranged from 1 to 100 μg/ml. Biological replicates for individual donors were excluded if a difference of > 10-fold was observed. Vertical bars represent the mean ± SEM.

## Slide 8
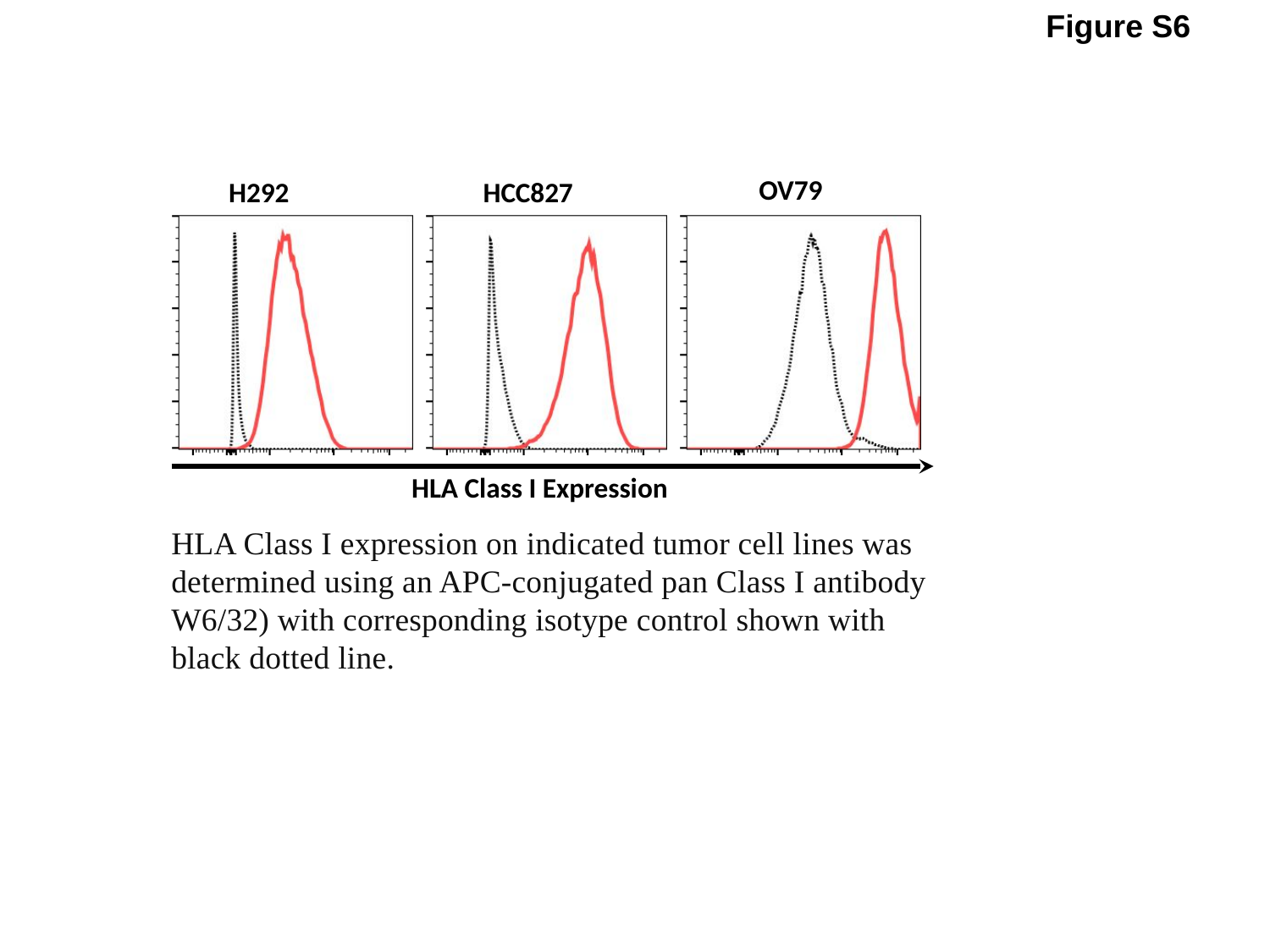

Figure S6
OV79
H292
HCC827
HLA Class I Expression
HLA Class I expression on indicated tumor cell lines was determined using an APC-conjugated pan Class I antibody W6/32) with corresponding isotype control shown with black dotted line.

## Slide 9
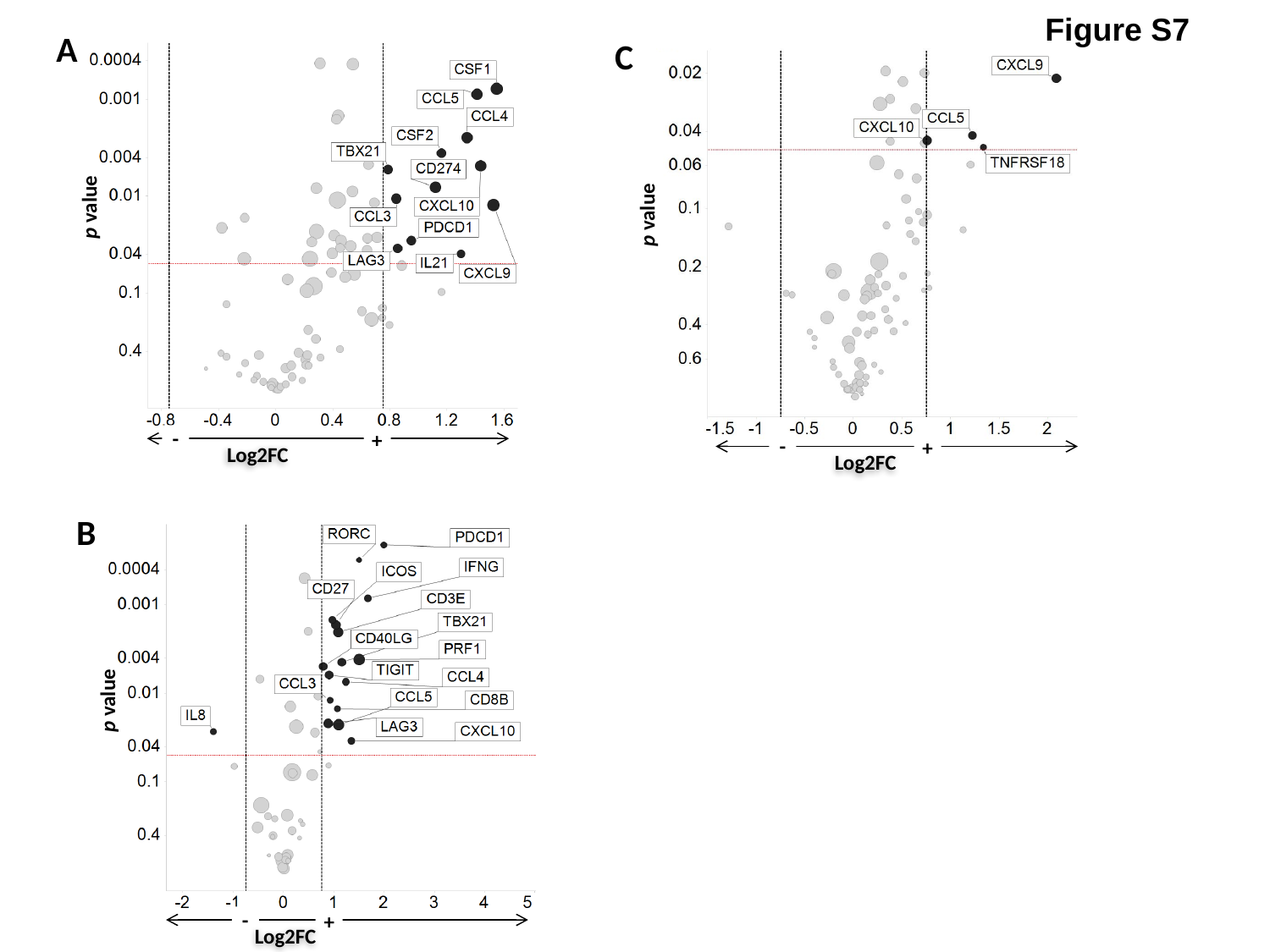

Figure S7
A
p value
-
+
Log2FC
C
p value
-
+
Log2FC
B
p value
-
+
Log2FC

## Slide 10
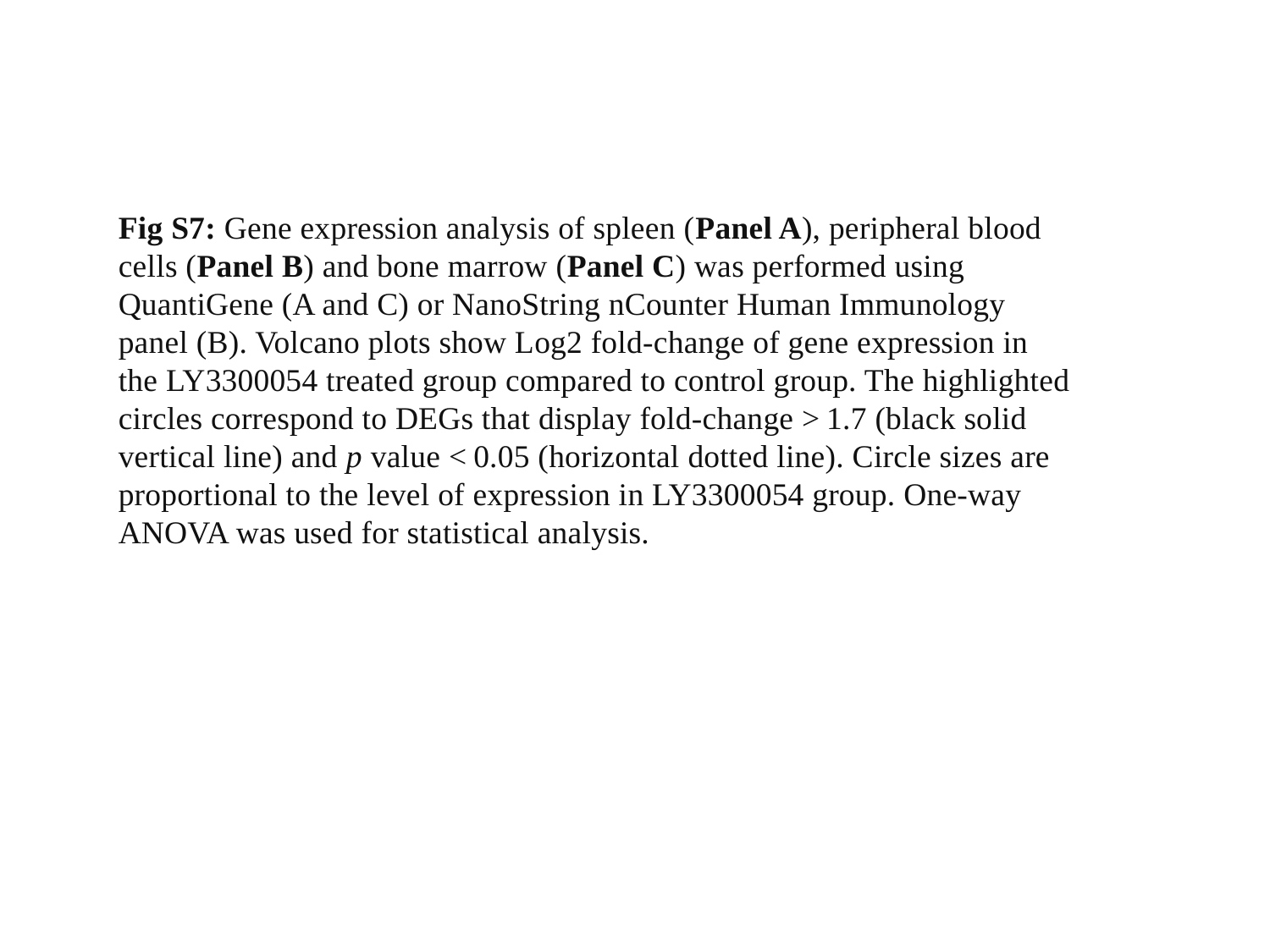

Fig S7: Gene expression analysis of spleen (Panel A), peripheral blood cells (Panel B) and bone marrow (Panel C) was performed using QuantiGene (A and C) or NanoString nCounter Human Immunology panel (B). Volcano plots show Log2 fold-change of gene expression in the LY3300054 treated group compared to control group. The highlighted circles correspond to DEGs that display fold-change > 1.7 (black solid vertical line) and p value < 0.05 (horizontal dotted line). Circle sizes are proportional to the level of expression in LY3300054 group. One-way ANOVA was used for statistical analysis.

## Slide 11
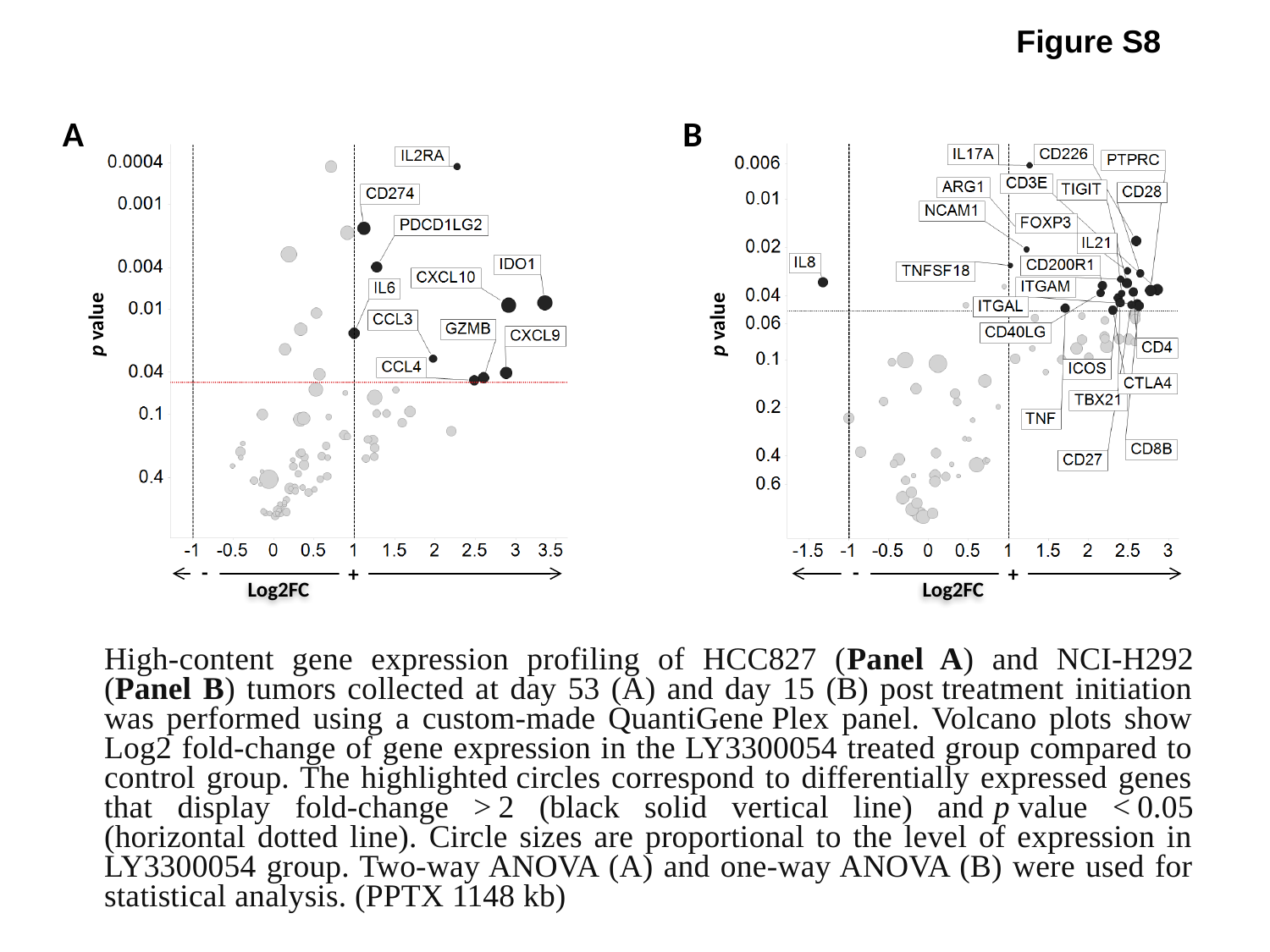

Figure S8
A
p value
-
+
Log2FC
B
p value
-
+
Log2FC
High-content gene expression profiling of HCC827 (Panel A) and NCI-H292 (Panel B) tumors collected at day 53 (A) and day 15 (B) post treatment initiation was performed using a custom-made QuantiGene Plex panel. Volcano plots show Log2 fold-change of gene expression in the LY3300054 treated group compared to control group. The highlighted circles correspond to differentially expressed genes that display fold-change > 2 (black solid vertical line) and p value < 0.05 (horizontal dotted line). Circle sizes are proportional to the level of expression in LY3300054 group. Two-way ANOVA (A) and one-way ANOVA (B) were used for statistical analysis. (PPTX 1148 kb)
