# Supplementary material for: Discovery and preclinical characterization of the antagonist anti-PD-L1 monoclonal antibody LY3300054
Source: J Immunother Cancer. 2018 Apr 30;6:31. doi: 10.1186/s40425-018-0329-7 (PMC5925824; doi:10.1186/s40425-018-0329-7)
Supplement: Supplementary file 2 — Supplementary Methods. (DOCX 34 kb) [file 40425_2018_329_MOESM2_ESM.docx]

**Additional File 1: Supplemental Methods**

*PD-L1 and HLA Class I staining of human tumor lines:*  NCI-H292, HCC827, OV79, and A204 (ATCC) tumor cells were cultured for approximately 36 hr prior to non-enzymatic harvest. NCI-H292, HCC827, and A204 cells were stained for PD-L1 with either a FITC-conjugated anti-human PD-L1 commercial antibody (clone MIH1, BD Biosciences), Alexa Fluor® 488-conjugated LY3300054, or appropriate isotype controls. NCI-H292, HCC827, and OV79 cells were stained separately for HLA Class I expression using an APC-conjugated antibody (clone W6/32, RnDSystems, Minneapolis, MN) Samples were collected on a 5-laser Fortessa X-20 cytometer (BD Biosciences) and analyzed with FlowJo V10 software (TreeStar).

**Milliplex Immunoassay**

*List of analytes*

EGF, Eotaxin, FGF-2, Flt-3L , Fractalkine, G-CSF , GM-CSF, GRO, IFNa , IFNg, IL-10, IL-12p40 , IL-12p70, IL-13, IL-15, IL-17a , IL-1a, IL-1b, IL-1RA, IL-2, IL-3 , IL-4, IL-5, IL-6 , IL-7, IL-8, IL-9, IP-10 , MCP-1, MCP-3, MDC , MIP-1a, MIP-1b, PDGF-aa, PDGFbb, RANTES, TGFa, TNFa, TNFb , VEGFA

**Quantigene Plex (QGP) Gene Expression Assay**

*List of targets*

*ARG1, B2M, C10ORF54, CCL2, CCL22, CCL3, CCL4, CCL5, CCR6, CD14, CD19, CD200R1, CD226, CD27, CD274, CD28, CD3E, CD4, CD40LG, CD68, CD8b, CEACAM8, CSF1, CSF2, CTLA4, CXCL10, CXCL11, CXCL8, CXCL9, CXCR3, DPP4, EEF1G, EIF4E2, EOMES, FAS, FOXP3, GATA3, GUSB, GZMB, HAVCR2, HLA-A, HLA-B, HLA-C, HLA-DRA, HPRT1, ICAM1, ICOS, IDO1, IFNA2, IFNB1, IFNG, IL10, IL15, IL17A, IL1B, IL2, IL21, IL23A, IL2RA, IL3RA, IL6, IL7, IL7R, ITGAL, ITGAM, ITGAX, LAG3, NCAM1, NOL7, NOS2A, OAS3, PAF1, PDCD1, PDCD1LG2, POLR2A, PPIB, PRF1, PSMB8, PTPRC, RORC, RPL19, RPS18, TBP, TBX21, TDO2, TGFB1, TGFB2, TGFB3, TIGIT, TNF, TNFRSF18, TNFRSF4, TNFRSF9, TNFSF18, TNFSF9*

**nCounter Gene Expression Assay**

*List of targets*

*ABCB1, ABL1, ADA, AHR, AICDA, AIRE, APP, ARG1, ARG2, ARHGDIB, ATG10, ATG12, ATG16L1, ATG5, ATG7, ATM, B2M, B3GAT1, BATF, BATF3, BAX, BCAP31, BCL10, BCL2, BCL2L11, BCL3, BCL6, BID, BLNK, BST1, BST2, BTK, BTLA, C14orf166, C1QA, C1QB, C1QBP, C1R, C1S, C2, C3, C4A/B, C4BPA, C5, C6, C7, C8A, C8B, C8G, C9, CAMP, CARD9, CASP1, CASP10, CASP2, CASP3, CASP8, CCBP2, CCL11, CCL13, CCL15, CCL16, CCL18, CCL19, CCL2, CCL20, CCL22, CCL23, CCL24, CCL26, CCL3, CCL4, CCL5, CCL7, CCL8, CCND3, CCR1, CCR10, CCR2, CCR5, CCR6, CCR7, CCR8, CCRL1, CCRL2, CD14, CD160, CD163, CD164, CD19, CD1A, CD1D, CD2, CD209, CD22, CD24, CD244, CD247, CD27, CD274, CD276, CD28, CD34, CD36, CD3D, CD3E, CD3EAP, CD4, CD40, CD40LG, CD44, CD46, CD48, CD5, CD53, CD55, CD58, CD59, CD6, CD7, CD70, CD74, CD79A, CD79B, CD80, CD81, CD82, CD83, CD86, CD8A, CD8B, CD9, CD96, CD97, CD99, CDH5, CDKN1A, CEACAM1, CEACAM6, CEACAM8, CEBPB, CFB, CFD, CFH, CFI, CFP, CHUK, CIITA, CISH, CLEC4A, CLEC4E, CLEC5A, CLEC6A, CLEC7A, CLU, CMKLR1, CR1, CR2, CRADD, CSF1, CSF1R, CSF2, CSF2RB, CSF3R, CTLA4_all (common probe), CTLA4-TM (membrane-bound form), sCTLA4 (soluble form), CTNNB1, CTSC, CTSG, CTSS, CUL9, CX3CL1, CX3CR1, CXCL1, CXCL10, CXCL11, CXCL12, CXCL13, CXCL2, CXCL9, CXCR1, CXCR2, CXCR3, CXCR4, CXCR6, CYBB, DEFB1, DEFB103A, DEFB103B, DEFB4A, DPP4, DUSP4, EBI3, EDNRB, EGR1, EGR2, ENTPD1, EOMES, ETS1, FADD, FAS, FCAR, FCER1A, FCER1G, FCGR1A/B, FCGR2A, FCGR2A/C, FCGR2B, FCGR3A/B, FCGRT, FKBP5, FN1, FOXP3, FYN, GATA3, GBP1, GBP5, GFI1, GNLY, GP1BB, GPI, GPR183, GZMA, GZMB, GZMK, HAMP, HAVCR2, HFE, HLA-A, HLA-B, HLA-C, HLA-DMA, HLA-DMB, HLA-DOB, HLA-DPA1, HLA-DPB1, HLA-DQA1, HLA-DQB1, HLA-DRA, HLA-DRB1, HLA-DRB3, HRAS, ICAM1, ICAM2, ICAM3, ICAM4, ICAM5, ICOS, ICOSLG, IDO1, IFI16, IFI35, IFIH1, IFIT2, IFITM1, IFNA1/13, IFNA2, IFNAR1, IFNAR2, IFNB1, IFNG, IFNGR1, IGF2R, IKBKAP, IKBKB, IKBKE, IKBKG, IKZF1, IKZF2, IKZF3, IL10, IL10RA, IL11RA, IL12A, IL12B, IL12RB1, IL13, IL13RA1, IL15, IL16, IL17A, IL17B, IL17F, IL18, IL18R1, IL18RAP, IL19, IL1A, IL1B, IL1R1, IL1R2, IL1RAP, IL1RL1, IL1RL2, IL1RN, IL2, IL20, IL21, IL21R, IL22, IL22RA2, IL23A, IL23R, IL26, IL27, IL28A, IL28A/B, IL29, IL2RA, IL2RB, IL2RG, IL3, IL32, IL4, IL4R, IL5, IL6, IL6R, IL6ST, IL7, IL7R, IL8, IL9, ILF3, IRAK1, IRAK2, IRAK3, IRAK4, IRF1, IRF3, IRF4, IRF5, IRF7, IRF8, IRGM, ITGA2B, ITGA4, ITGA5, ITGA6, ITGAE, ITGAL, ITGAM, ITGAX, ITGB1, ITGB2, ITLN1, ITLN2, JAK1, JAK2, JAK3, KCNJ2, KIR_Activating_Subgroup_1, KIR_Activating_Subgroup_2, KIR_Inhibiting_Subgroup_1, KIR_Inhibiting_Subgroup_2, KIR3DL1, KIR3DL2, KIR3DL3, KIT, KLRAP1, KLRB1, KLRC1, KLRC2, KLRC3, KLRC4, KLRD1, KLRF1, KLRF2, KLRG1, KLRG2, KLRK1, LAG3, LAIR1, LAMP3, LCK, LCP2, LEF1, LGALS3, LIF, LILRA1, LILRA2, LILRA3, LILRA4, LILRA5, LILRA6, LILRB1, LILRB2, LILRB3, LILRB4, LILRB5, LITAF, LTA, LTB4R, LTB4R2, LTBR, LTF, LY96, MAF, MALT1, MAP4K1, MAP4K2, MAP4K4, MAPK1, MAPK11, MAPK14, MAPKAPK2, MARCO, MASP1, MASP2, MBL2, MBP, MCL1, MIF, MME, MR1, MRC1, MS4A1, MSR1, MUC1, MX1, MYD88, NCAM1, NCF4, NCR1, NFATC1, NFATC2, NFATC3, NFIL3, NFKB1, NFKB2, NFKBIA, NFKBIZ, NLRP3, NOD1, NOD2, NOS2, NOTCH1, NOTCH2, NT5E, PAX5, PDCD1, PDCD1LG2, PDCD2, PDGFB, PDGFRB, PECAM1, PIGR, PLA2G2A, PLA2G2E, PLAU, PLAUR, PML, POU2F2, PPARG, PPBP, PRDM1, PRF1, PRKCD, PSMB10, PSMB5, PSMB7, PSMB8, PSMB9, PSMC2, PSMD7, PTAFR, PTGER4, PTGS2, PTK2, PTPN2, PTPN22, PTPN6, PTPRC_all (common probe), CD45R0, CD45RA, CD45RB, PYCARD, RAF1, RAG1, RAG2, RARRES3, RELA, RELB, RORC, RUNX1, S100A8, S100A9, S1PR1, SELE, SELL, SELPLG, SERPING1, SH2D1A, SIGIRR, SKI, SLAMF1, SLAMF6, SLAMF7, SLC2A1, SMAD3, SMAD5, SOCS1, SOCS3, SPP1, SRC, STAT1, STAT2, STAT3, STAT4, STAT5A, STAT5B, STAT6, SYK, TAGAP, TAL1, TAP1, TAP2, TAPBP, TBK1, TBX21, TCF4, TCF7, TFRC, TGFB1, TGFBI, TGFBR1, TGFBR2, THY1, TICAM1, TIGIT, TIRAP, TLR1, TLR2, TLR3, TLR4, TLR5, TLR7, TLR8, TLR9, TMEM173, TNF, TNFAIP3, TNFAIP6, TNFRSF10C, TNFRSF11A, TNFRSF13B, TNFRSF13C, TNFRSF14, TNFRSF17, TNFRSF1B, TNFRSF4, TNFRSF8, TNFRSF9, TNFSF10, TNFSF11, TNFSF12, TNFSF13B, TNFSF15, TNFSF4, TNFSF8, TOLLIP, TP53, TRAF1, TRAF2, TRAF3, TRAF4, TRAF5, TRAF6, TYK2, UBE2L3, VCAM1, VTN, XBP1, XCL1, XCR1, ZAP70, ZBTB16, ZEB1, ABCF1, ALAS1, EEF1G, G6PD, GAPDH, GUSB, HPRT1, OAZ1, POLR1B, POLR2A, PPIA, SDHA, TBP, TUBB, RPL19,*
